# Emotion-specific amygdala habituation in treatment-referred young adults with antisocial histories: links with internalizing and externalizing symptoms and future recidivism

**DOI:** 10.1016/j.ynirp.2026.100371

**Published:** 2026-06-11

**Authors:** Ilse H. van de Groep, Josjan Zijlmans, Reshmi Marhe

**Affiliations:** a Erasmus School of Social and Behavioral Sciences, Erasmus University Rotterdam, the Netherlands; b Center for Substance use and Addiction Research, Erasmus University Rotterdam, the Netherlands; c Department of Child and Adolescent Psychiatry, Amsterdam University Medical Center, Amsterdam, the Netherlands

**Keywords:** Habituation, Externalizing, Internalizing, Recidivism, Amygdala, fMRI

## Abstract

**Background:**

Habituation reduces the salience of repeated socio-emotional cues like facial expressions once they become familiar, predictable, or no longer relevant. To clarify whether variation in amygdala habituation to repeated emotional faces is associated with psychological symptoms and persistent antisocial behavior, the current study examined whether internalizing and externalizing symptoms, and their interaction, were related to amygdala habituation in treatment-referred young adults with antisocial histories, and whether habituation predicted future recidivism.

**Methods:**

For this purpose, ninety-eight treatment-referred young adults (18-27) with a history of antisocial behavior performed an emotional face-matching fMRI task presenting fearful, angry, sad, happy and neutral faces. Habituation was operationalized as the change in amygdala activation from the first to the second half of the task for each emotional face condition. Internalizing and externalizing symptoms were measured with the Adult Self-Report. Recidivism was obtained from Dutch national judicial records (median follow-up 2.5 years post-scan).

**Results:**

Amygdala habituation differed by emotional expression in the treatment-referred youth, with the strongest habituation for happy faces and relatively weaker habituation for negative and neutral expressions. However, within this group, individual differences in habituation were not associated with internalizing symptoms, externalizing symptoms, their interaction or recidivism. Additional analyses comparing treatment-referred youth with healthy controls indicated that treatment-referred young adults showed reduced habituation to fearful faces compared to controls.

**Conclusion:**

Taken together, these findings suggest that amygdala habituation may help identify emotion-specific neural processing differences between treatment-referred young adults and controls, but is not associated with dimensional symptom heterogeneity or future recidivism within treatment-referred young adults.

## Highlights

• 98 multi-problem young adults completed an emotional face-matching fMRI task.
• Amygdala habituation was strongest for happy faces.
• Internalizing symptoms amplified externalizing-related recidivism risk.
• Amygdala habituation did not predict or moderate recidivism risk.

## 1 Introduction

During the transition from adolescence to adulthood, antisocial behavior may persist or change as young adults navigate a rapid succession of challenges (Arnett, 2000; Zijlmans et al., 2021a, Zijlmans et al., 2021b), such as identity exploration, and alignment of personal goals with increasing societal demands and rapidly changing social roles, norms and contexts (Arnett, 2000; Nelson, 2021; van de Groep et al., 2023). To face these challenges, young adults need to learn and optimize the ability to *habituate*. During this non-associative learning process, individuals learn to show reduced behavioral or neural responsivity to social and emotional stimuli they are repeatedly exposed to (McDiarmid et al., 2017; Plichta et al., 2014; Ramaswami, 2014; Rankin et al., 2009). For instance, upon entering a new peer group, young adults may be highly sensitive to neutral social feedback or delayed replies, but if these cues re-occur without negative consequences such as rejection or conflict, they will over time evoke less intense responding. Such cues can become irrelevant due to their familiarity, predictability or lack of negative consequences (Bas-Hoogendam et al., 2019; McDiarmid et al., 2017; Plichta et al., 2014; Ramaswami, 2014; Rankin et al., 2009). Building on this idea, the current study examined whether habituation to emotional facial expressions, measured by amygdala responses to repeatedly presented emotional faces, is associated with internalizing and externalizing symptoms and future recidivism in multi-problem young adults.

### 1.1 Neural habituation to socio-emotional cues

One way to study habituation is to examine how reactive the brain is in response to novel social and emotional stimuli (e.g., emotional faces), and whether this response habituates over time (Bas-Hoogendam et al., 2019). In typically developing individuals, such repeated presentation of social and emotional cues during fMRI tasks results in decreased neural activation over time (Kleinhans et al., 2016), which is thought to reflect adaptive, efficient processing of socio-emotional information (Bas-Hoogendam et al., 2019; Berhe et al., 2023; Blackford et al., 2013; Gerhardt et al., 2023; Kleinhans et al., 2009, 2009, 2009; Ramaswami, 2014)Importantly, fMRI signals of habituation may be particularly relevant to understand individual differences in emotional processing, since they capture whether individuals update and down-regulate responses to socio-emotional cues, by measuring change after repeated exposure to stimuli, rather than average response magnitude. Prior work using emotional face processing paradigms showed that amygdala habituation has higher test-retest reliability than mean response amplitude, suggesting that it may reflect a more reliable measure than mean level activity (Elliott et al., 2020; Kragel et al., 2021; Plichta et al., 2014).

### 1.2 Clinical relevance of amygdala habituation

Habituation can also be considered clinically relevant, since it signals how salience of social information is updated if social and emotional cues are repeated (McDiarmid et al., 2017, 2019; Stevens et al., 2021). The amygdala usually rapidly habituates to socio-emotional stimuli (Plichta et al., 2014), whereas slower habituation or increased sensitization has been reported across various internalizing and externalizing classifications characterized by atypical social behavior such as Autism Spectrum Disorder (ASD) (Kleinhans et al., 2009, 2016; Swartz et al., 2013; Wiggins et al., 2014), Social Anxiety Disorder (SAD) (Bas-Hoogendam et al., 2019; Sladky et al., 2012), Post Traumatic Stress Disorder (PTSD) (Hinojosa et al., 2023), Borderline Personality Disorder (BPD) (Bilek et al., 2019) and Substance Use Disorders (Gerhardt et al., 2023; Regier et al., 2022). Second, slower habituation has been linked to higher symptom severity (Hinojosa et al., 2023; McDiarmid et al., 2017, 2019; although not always, see e.g., Bilek et al., 2019).

As such, decreased amygdala habituation may reflect a trans-diagnostic neuro-affective variation in the extraction and interpretation of social and emotional information (Adolphs, 2010; Kleinhans et al., 2016; Schettini et al., 2021; Tam et al., 2017), and/or variations in salience and arousal detection in the socio-emotional information (Kleinhans et al., 2016; Stevens et al., 2023). However, the current studies that examine the association between amygdala and mental health symptoms habituation have several limitations, that hamper progress in establishing whether amygdala habituation can indeed serve as a transdiagnostic neuro-affective mechanism in young adults (Beauchaine and Hinshaw, 2020; Schettini et al., 2021).

First, a growing body of research suggests that mental health problems are continuous (Beauchaine and Hinshaw, 2020; Dalgleish et al., 2020; Krueger et al., 2018), but this dimensional nature of mental health issues is often not addressed in research on amygdala habituation (but see Berhe et al., 2023). Thus, it is important to assess whether amygdala habituation is associated with more dimensional measures of mental health symptoms, such as internalizing and externalizing symptoms (Achenbach et al., 2016; Stevens et al., 2021).

Second, internalizing and externalizing symptoms often co-occur within individuals, and this co-occurrence can either amplify externalizing problems (Dalgleish et al., 2020; Schettini et al., 2021; Shi et al., 2020; Viding et al., 2024; Berhe et al., 2023). or serve as a protective factor for youth with externalizing problems (Beauchaine and Thayer, 2015), resulting in better social adaptation (e.g., showing less aggression and having better peer relationships, see Schettini et al., 2021). Thus, when examining the association between amygdala habituation and mental health problems, it is important to consider and statistically model the interaction between internalizing and externalizing problems to account for potential functional dependencies (Beauchaine and Hinshaw, 2020; Berhe et al., 2023; Schettini et al., 2021; Stevens et al., 2023; Viding et al., 2024). Therefore, the first aim of the current study is to examine whether (interacting) levels of internalizing and externalizing problems are associated with amygdala habituation in treatment-referred young adults (i.e., with co-occurring externalizing and internalizing problems, a history of delinquency, lack of (sufficient) income, etc.) (Zijlmans et al., 2021).

**Amygdala habituation as a predictor of future recidivism** Third, although prior studies have examined correlates of amygdala habituation (e.g. social support such as *maternal care* (Holz et al., 2021; Stevens et al., 2023); *genetic influences*, (Wiggins et al., 2014)*; Adverse Childhood Experiences (ACEs)* (Bilek et al., 2019; Gerhardt et al., 2023; Kim et al., 2019; Stevens et al., 2023), much less is known about whether altered amygdala habituation is also associated with externalizing behavior in daily life. This is surprising, given that several researchers have theorized that amygdala habituation is associated with impairments in social (e.g., Kleinhans et al., 2016; Gerhardt et al., 2023; Bas-Hoogendam et al., 2019; Blackford et al., 2013), emotional (Berhe et al., 2023) and adaptive functioning in general (McDiarmid et al., 2017). Therefore, in the current study we test whether individual differences in amygdala habituation are associated with recidivism, which may signal persistent antisocial behavior in daily life.

In youth with a history of antisocial behavior, habituation to socio-emotional cues may be altered in at least two ways: through *sustained engagement with threat-related cues* or *increased disengagement* from social and emotional cues. Both patterns may be relevant to persistent antisocial behavior or recidivism: sustained engagement with threat-related cues may increase vulnerability to reactive or defensive responses (Bertsch et al., 2020), whereas disengagement from social and emotional cues may reduce sensitivity to the consequences of one's behavior for others (Zhao et al., 2025). Sustained engagement with threat-related cues). , which may likely optimizes the efficiency of neural circuits for future threat detection in social contexts (Hardi et al., 2025; Nikolic et al., 2022). Although this could make individuals more responsive to future threat-related cues, it may also increase vulnerability to stressors, reduce one's ability to process other aspects of the social situation, and de-prioritize updating the relevance of repeated social signals (Hardi et al., 2025). In line with this idea, Presentation of angry faces, which typically signal hostility and thus social threat (Bertsch et al., 2020), tend to elicit hyperactivation in the right amygdala in antisocial individuals (Coccaro et al., 2007; Jeung-Maarse et al., 2023; McCloskey et al., 2016 but see Passamonti et al., 2010). Research likewise shows that individuals with externalizing behavior may interpret neutral and fearful faces as ambiguous threats that require increased attentional monitoring (Bertsch et al., 2020; Brennan et al., 2018; van de Groep et al., 2022, 2023). This pattern is also consistent with the concept of hostile attribution bias (Dodge et al., 1990): the socio-cognitive tendency to interpret ambiguous cues as hostile, which is often observed in youth with a history of antisocial behavior (Klein Tuente et al., 2019).

As mentioner earlier a lack of habituation can also occur due to *increased disengagement* from social and emotional cues. If emotional cues remain aversive or lose social relevance, persistent disengagement could arise, which could disrupt learning from such cues (Ellis et al., 2011, 2022). Such disengagement fits well with earlier empirical findings in youth with antisocial behavior, such as a reduced ability to respond to distress in others that is observed in individuals with antisocial behavior (Zhao et al., 2025), and the corresponding failure to internalize potential negative consequences of aggressive acts (van de Groep et al., 2023).In line with this idea, several studies in antisocial populations report hypoactivation of the right or bilateral amygdala in response to fearful faces (Berluti et al., 2023; Dotterer et al., 2017; Jones et al., 2009; Sebastian et al., 2021)and sad faces (Passamonti et al., 2010). Taken together, this suggests that for fearful and sad faces, reduced habituation may either occur due to (1) a high neural sensitivity that is not desensitized (Coccaro et al., 2007; Jeung-Maarse et al., 2023; McCloskey et al., 2016), or (2) due to stable and low levels of amygdala activity that reflect disengagement (Berluti et al., 2023; Dotterer et al., 2017; Jones et al., 2009; Sebastian et al., 2021)), highlighting the need to examine whether habituation depends on the emotional stimulus type. For positively valenced faces, such as happy faces, there is no evidence for altered amygdala reactivity in antisocial populations (Jeung-Maarse et al., 2023).

Few studies have investigated whether amygdala functioning predicts recidivism (Jansen, 2022; Nikolic et al., 2022; van Dongen et al., 2024). Although prior work (e.g. Dotterer et al., 2017) has linked amygdala reactivity to angry and fearful faces to recidivism, it remains unclear whether habituation has a similar predictive value (Plichta et al., 2014), across different types of facial expressions (e.g. angry, fearful, sad, neutral, happy).

### 1.3 The current study

In the current study, we used a dimensional approach to examine amygdala habituation to emotional faces in treatment-referred young adults with histories of antisocial behavior. We tested whether habituation varied across emotional expressions and whether individual differences in habituation were associated with internalizing symptoms, externalizing symptoms, and their interaction. While we hypothesized *that* internalizing and externalizing problems would interactively predict amygdala habituation (Dalgleish et al., 2020; Schettini et al., 2021; Shi et al., 2020), previous literature points towards two opposing hypotheses for the *direction* of this effect: the influence of internalizing problems may either strengthen (Shi et al., 2020; Berhe et al., 2023) or weaken (Beauchaine and Thayer, 2015; Schettini et al., 2019) the effects of externalizing behavioral problems on amygdala habituation. We also explored whether the direction and strength these effects depend on the type of emotional expression (Jeung-Maarse et al., 2023; McCloskey et al., 2016; Coccaro et al., 2007; Dotterer et al., 2017; Jones et al., 2009; Sebastian et al., 2021; Dotterer et al., 2017, 2017; Berluti et al., 2023). Additional group comparisons with controls were included to contextualize the habituation patterns, but the primary focus remained on individual differences within the treatment-referred group.

Our second aim was to examine whether habituation was prospectively associated with recidivism. We hypothesized that amygdala habituation would negatively predict overall and serious recidivism (Dotterer et al., 2017), beyond factors that have been identified in the literature such as ACEs (Yohros, 2023), substance use (Hoeve et al., 2013; McReynolds et al., 2010; Pankow et al., 2024; Robertson et al., 2020; van der Put et al., 2014) and prior offending (Cottle et al., 2001), and that the interaction with internalizing and externalizing could either strengthen (Shi et al., 2020; Berhe et al., 2023) or weaken (Beauchaine and Thayer, 2015; Schettini et al., 2019) this effect.

Although the primary focus of this study, in line with the two aims, is on region-of-interest (ROI) analyses in multi-problem young adults, we also report behavioral (e.g., accuracy and reaction times) and whole brain analyses in both the multi-problem group and a gender-matched control group. These additional analyses are intended to provide a broader characterization of task performance and neural activation, and to situate the ROI findings within the wider task context. Because data on internalizing problems, externalizing problems, and recidivism were only available for the multi-problem subgroup, the main hypothesis-driven analyses were restricted to this group.

## 2 Methods

### 2.1 Participants

127 young adult males aged between 18 and 27 (M = 22, SD = 2.5) participated in the current study. For the fMRI study, 102 participants were drawn from a larger longitudinal cohort of 696 young adults referred to De Nieuwe Kans, a Rotterdam-based day-treatment program for young adults with co-occurring psychosocial, psychiatric, substance-use, and justice-related problems (see Luijks et al., 2017; van Duin et al., 2019; Van Duin et al., 2021; Zijlmans et al., 2021; Zijlmans et al., 2018; Zijlmans et al., 2021 for more detailed descriptions in prior publications). (;. These treatment-referred young adults with a history of antisocial behavior (note that in the literature these are sometimes also referred to as “multi-problem” young adults (see e.g. Zijlmans et al., 2021a, Zijlmans et al., 2021b; Luijks et al., 2017) showed high levels of internalizing and externalizing problems relative to same-aged norms (see Table 1 and section 2.2.1.2). For the fMRI study, 25 gender-matched control young adult participants (∼20% of the total sample) were recruited via online flyers and through visits to vocational education colleges (MBO) with the aim to recruit participants in the same age range and with a similar average educational level as the treatment-referred young adult group. Young adults in the control group were slightly older than those in the treatment-referred group and showed a lower number of cannabis use years than those in the treatment-referred group (see Table 1 and section 2.2.2.4). In addition, the distribution of ethnicity differed significantly between groups (see Table 1 and section 2.2.2.1 for more details).

**Table 1:** Sample description and group comparisons.

Measure	Multi-problem Young Adults (n = 98)	Control Group (n = 25)	Statistical comparison	Cohen's d [95% CI]
Age (years) [*M* (*SD*)]	21.77 (2.37)	23.10 (2.61)	t(36.68) = 2.367, p = .023	0.537 [0.072, 0.994]
Cannabis use (years) [*M* (*SD*)]	4.22 (3.82	1.40 (2.63)	t(54.0) = −4.30 (54.0), p < .001	−0.86 [-1.28, −0.43]

**Measure**			**Statistical comparison**	**Effect size (Cramér's V [95% CI])**

Ethnicity			χ^2^ (Monte Carlo^1^)(8) = 71.57, p < .001	0.76 [0.51, 1.00]
	**Multi-problem Young Adults (n = 98)**	**Control Group (n = 25)**	**Δ pp (Multiproblem vs. Control)**	***p* (Holm)^2^**

Dutch	14.3%	33.3%	−19.0	**.024**
Antillean	27.6%	0%	27.6	**.002**
Morroccan	14.3%	0%	14.3	**.037**
Turkish	2.0%	11.1%	−9.1	**.033**
Cape Verdean	7.1%	0%	7.1	0.153
Surinamese	16.3%	3.7%	12.6	0.09
Other Western	2.0%	18.5%	−16.5	**< .001**
Other non-Western	16.3%	0%	16.3	**.025**
Unknown/Other	0%	33.3	−33.3	**< .001**

**Measure**	**Multi-problem Young Adults (n = 98)**			

Internalizing Problems (ASR) [*M* (*SD*)]	73.6 (23.1)			
Externalizing Problems (ASR) [*M* (*SD*)]	70.5 (22.8)			
DSM Antisocial personality problems clinical range (ASR)	52% Not clinical			
	15.3% Borderline clinical			
	32.7% Clinical			
Number of ACEs [*M* (*SD*)]	3.8 (1.9)			
IQ Score [*M* (*SD*)]	82.2 (10.6)			
Number of participants with a prior criminal record [%]	55% no			
	45% yes			
Amount of prior criminal offenses [*M* (*SD*)]	4 (7.6)			

1 Monte Carlo p-value based on 20,000 replicates.

2 p-values are Holm-adjusted for multiple comparisons across categories. Bold = significant at α = .05.

All participants had normal or corrected-to-normal vision and were screened on fMRI contraindications prior to participation. The study protocol was approved by the VU University Medical Center Medical Ethical Committee (registration number 2013.422–NL46906.029.13) and in accordance with the Declaration of Helsinki, all participants provided informed consent. Participants received a financial reimbursement for their participation (€30).

### 2.2 Materials

#### 2.2.1 Baseline measures

##### 2.2.1.1 Procedure and emotional face processing task

**Fig. 1:**
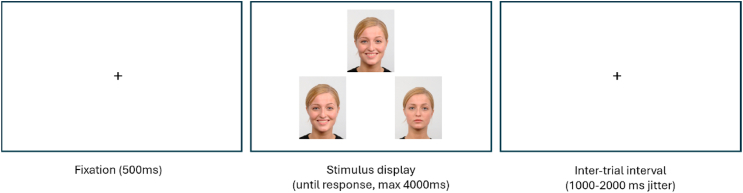
Schematic depiction of a trial of the emotional face-processing task.

Participants performed an emotional face-matching task (Hariri et al., 2002) (see Fig. 1), selected because it reliably elicits amygdala reactivity (Dotterer et al., 2017; Hariri et al., 2002) and habituation (Gerhardt et al., 2023; Plichta et al., 2014). During the task, participants are presented with three stimuli (faces or shapes) in a left, middle and right position, and subsequently asked to match either a face or a shape to an identical target stimulus of the same category. The face stimuli were neutral, happy, angry, sad and fearful faces, and the shapes were triangles, ovals and squares. Compared to the original emotion recognition task design (Hariri et al., 2002), we added three more emotion categories (sadness, happy, neutral), which thus includes a within-category baseline (neutral), and allowed us to examine whether habituation effects were specific to threat, positive valence, or negative valence. Moreover, instead of the Ekman facial expression stimuli used in the original task, we used facial expression stimuli from the Radboud Faces Database (Langner et al., 2010).

In total, participants completed 1 run (lasting 10 min) consisting of 120 face trials (24 trials per emotional face type) and 24 shape trials (8 trials per shape) presented in a semi-randomized manner. The run was structured in mini-blocks, where participants completed short blocks of four consecutive trials from the same face condition, interleaved with shape-matching control blocks containing geometric stimuli. Note that although the presentation was mini-blocked, first-level analyses modeled condition-specific events separately to estimate early and late responses for each emotional expression. Each trial started with a fixation cross (500ms), which was followed by a face or shape stimulus (until response with a max. of 2000ms), followed by jitter ranging from 1200 to 2000ms).

##### 2.2.1.2 Internalizing and externalizing problems

Internalizing and Externalizing problems in multi-problem young adults were assessed using the ASEBA Adult Self Report (Achenbach et al., 2003, 2016). Individuals indicated whether 123 statements applied to them in the past six months on a three-point Likert scale: 0 (‘Not true’), 1 (‘Somewhat or sometimes true’), 2 (‘Very true or often true’), with higher scores indicating higher severity of problem behavior. From these items, sex- and gender-based t-scores were calculated for the Internalizing and Externalizing scales. T-scores above 64 are generally considered to reflect ‘Clinical” problem levels. Mean scores for the treatment-referred group fell above this level for both the Internalizing and Externalizing subscales (see Table 1). Cronbach's alpha was 0.88 for Internalizing and 0.89 for Externalizing. Note that Internalizing and Externalizing problems were not assessed in the Control group.

#### 2.2.2 Covariates

##### 2.2.2.1 Demographic characteristics

Demographic characteristics assessed at baseline included age and ethnicity. Ethnicity classification was based on the birth country of the participants and their parents, according to the Dutch *Centraal Bureau voor de Statistiek* definition divided into 8 categories: Dutch, Moroccan, Antillean, Surinamese, Cape Verdean, Turkish, other Western, and other non-Western (van Duin et al., 2019; Zijlmans et al., 2021).

##### 2.2.2.2 Adverse Childhood Experiences

Adverse childhood experiences (ACEs) were assessed using two self-report questionnaires where participants indicated whether they had been exposed to 11 types of ACEs falling within three overarching categories: abuse (3: physical, emotional, sexual), neglect (2: physical, emotional), and household dysfunction (6: alcohol abuse in the family, drug abuse in the family, police contact in the family, psychological problems in the family, domestic violence, and growing up in a single-parent family) (van Duin et al., 2019, 2020). For abuse and neglect, the 24-item Dutch Childhood Trauma Questionnaire–Short Form (CTQ-SF) was used. For household dysfunction six single items were used, (see van Duin et al., 2019 for a more elaborate description). A total ACE score across the first 18 years of life was computed to closely match the categorization used in the ACE study (Dong et al., 2004; see van Duin et al., 2019, 2020 for a more elaborate description).

##### 2.2.2.3 Criminal history

Participants’ criminal history was determined based on the judicial documentation provided by the Dutch Ministry of Security and Justice and operationalized as the number of previous convictions for offenses prior to the baseline measurements (van Duin et al., 2019, 2021).

##### 2.2.2.4 Cannabis use

To assess years of regular cannabis use, participants completed the Addictions for Triage and Evaluation Questionnaire (MATE) (Schippers et al., 2010; Zijlmans et al., 2021; van Duin et al., 2019). Cannabis use was the most frequently reported primary problem substance in the current sample (28.6%). Regular cannabis users reported use for 15.2 days (SD = 13.2) on average out of the last 30 days.

#### 2.2.3 Follow-up measures

##### 2.2.3.1 Recidivism

Recidivism data of treatment-referred young adults was obtained from judicial documentation provided by the Dutch Ministry of Security and Justice. This documentation indicated whether an individual was arrested for a criminal offense between the start of the baseline measurements and January 2018, as well as the time to arrest for criminal offenses. Based on the definition of the Dutch Ministry of Security and Justice, the arrest and time to arrest could be classified as *general* (i.e., occurrence of - and time to any type of criminal offense, including both minor offenses (e.g. possession of a weapon) and serious offenses such as assault, rape and aggravated theft) or *serious* (i.e., occurrence of - and time to serious offenses with a minimum 4-year sentence (Zijlmans et al., 2021). The median follow-up time to arrest was 18 months (range = <1-40 months). Out of the 98 participants, 53 individuals showed recidivism (63.1%), 31 participants did not show any recidivism (36.9%), and 14 individuals had missing data (14.4%). Note that recidivism was not assessed in the Control group.

### 2.3 Neuroimaging methods

#### 2.3.1 MRI data acquisition

MRI data was acquired using a 3T GE Healthcare MRI scanner with a standard head coil at Erasmus Medical Center Rotterdam. For functional MRI scans, blood oxygen level-dependent T2*-weighted images Functional images were acquired in the axial plane using a gradient-echo echo-planar imaging sequence (repetition time = 2 s, echo time = 30msec, flip angle = 80°, sequential acquisition: 42 slices, voxel size = 3.44 × 3.44 × 3.5 mm, 64 × 64 matrix, field of view = 220 mm). In addition to the fMRI sequences, anatomical reference images were acquired using a fast-spoiled gradient echo sequence comprising 180 consecutive sagittal slices., with a thickness of 1.0 mm (repetition time = 6.4 ms, echo time = 2.8 ms, flip angle = 12°, 3D matrix size for 3D acquisitions: 240 × 240 slices, voxel size = 1 × 1 × 1 mm, field of view = 240 mm). The scanner automatically discarded dummy scans for stabilization.

#### 2.3.2 Preprocessing

Functional imaging data were analyzed using Statistical Parametric Mapping 12 (SPM12; Wellcome Department of Cognitive Neurology, London, United Kingdom). Images were realigned, corrected for slice timing, spatially normalized to MNI space using T1-based templates, resampled to 3 × 3 × 3 mm voxels, and smoothed with a 6-mm FWHM Gaussian kernel. Participants were retained for first-level analyses if they completed the task and showed less than 3 mm of head motion in each translation direction. Five participants were excluded because they did not complete the task (n = 2) or exceeded the motion threshold (n = 3), resulting in a final fMRI sample of 122 participants.

#### 2.3.3 First level analyses and habituation index

To perform statistical analyses on individual subjects' fMRI data, the general linear model in SPM12 was used. We modeled the fMRI time series as a series of zero-duration events with a canonical hemodynamic response function, using ‘Neutral-Early, ‘Neutral-Late’, ‘Angry-Early’, ‘Angry-Late’, ‘Sad-Early, ‘Sad-Late’, ‘Fearful-Early’, ‘Fearful-Late’, ‘Happy-Early’ and ‘Happy-Late’ as regressors for the face-processing part of the task. The difference between “Early” and “Late” was based on the well-established habituation index (i.e., the difference between the first (“Early”) and second half (“Late) of the experimental task). For the control condition, we only modeled two related events: “Control-Early” and “Control-Late” (i.e. not divided into separate contrasts per shape). Trials with no response were modeled separately as a regressor of no interest and were excluded from analyses. Six movement parameters were added as covariates of no interest. The pairwise comparisons led to participant-specific contrast images, which were subsequently submitted to second-level group analyses.

#### 2.3.4 Second level analyses

We performed a full factorial analysis of variance (ANOVA) with face condition (5: Neutral, Angry, Sad, Fearful, Happy) and time (Early vs. Late) to examine whole brain neural habituation effects across different types of emotional faces. More specifically, we computed and tested the aggregated contrasts “Faces vs. Control”, “Late Faces vs. Early Faces”; as well as the emotion-specific contrasts “Late Neutral vs. Early Neutral”, “Late Angry vs. Early Angry”, “Late Sad vs. Early Sad”, “Late Fearful vs. Early Fearful”, “Late Happy vs. Early Happy”. All results were corrected using an FDR cluster-corrected threshold of p < .001. Local maxima coordinates are reported using MNI space.

#### 2.3.5 Region-of-interest analyses (ROIs)

To test our hypotheses about the (1) interactive effect of internalizing and externalizing problems on amygdala habituation and (2) predictive effect of amygdala habituation on recidivism, we created 2 ROIs using the MarsBaR toolbox for SPM12 (Brett, Anton, Valabregue, and Poline, 2002), by extracting parameter estimates for the left amygdala (x = −24, y = 4, z = −18) and right amygdala (x = 24, y = −4, z = −18) We extracted parameter estimates from 5-mm spherical ROIs centered on bilateral amygdala coordinates selected to approximate the anatomical center of the AAL-defined amygdala region (AAL; Tzourio-Mazoyer et al., 2002).

#### 2.3.6 Statistical analyses

Behavioral and ROI data were analyzed using R (Version 4.0.1, R Core team, 2020). For task performance analyses (Accuracy and Reaction time), we included the same fMRI sample as specified earlier (n = 122; n = 98 treatment-referred young adults and n = 25 controls). Given that data on Internalizing and Externalizing problems, as well as Recidivism was only available for the treatment-referredsub-sample (n = 98), our main hypotheses were tested in this subgroup only.

##### 2.3.6.1 The effect of internalizing and externalizing problems on amygdala habituation

To test our first hypothesis about the interactive effect of internalizing and externalizing problems on amygdala habituation, we fitted several linear mixed models using REML estimation with random intercepts and slopes for time by subject,^1^ for left and right amygdala parameters estimates separately. The baseline model included fixed effects for Time, Condition, their interaction, all covariates (History of delinquency, ACEs Total Score, Cannabis use, Age, Ethnicity and Habituation type), and random intercepts and slopes for time. We then tested whether adding the Internalizing × Externalizing × Time interaction, and the Internalizing × Externalizing × Time × Condition term improved the model fit (Brown, 2021; Meteyard and Davies, 2020).

We decided to run separate models for each ROI after visual inspection and comparison of ROI difference scores between Early and Late revealed that some individuals showed opposing patterns of habituation/sensitization for the left and right amygdala. To account for multiple comparisons, while also accounting for the correlation between left and right amygdala ROI estimates (R = 0.297), we applied a modified Bonferroni correction adjusted for correlated variables (Sankoh et al., 1997) of α = .05/1.703281 = 0.0294. The covariate habituation type (habituation vs. sensitization) was added to the model to account for individual differences in amygdala response trajectories over time. Including this covariate helped reduce potential suppression effects that could arise when opposite response patterns are analyzed together, as habituation and sensitization may partially cancel each other out at the aggregate level. For both models, Satterthwaite's method was used to approximate degrees of freedom. With respect to the model assumptions, for both the left and right amygdala, we identified univariate ROI outliers, which were subsequently winsorized (Tabachnick and Fidell, 2013). Results were comparable before and after winsorization; therefore, the winsorized results are reported here.

Note that Internalizing and externalizing symptoms were moderately correlated (r = 0.66, 95% CI [0.54, 0.76]). Because our research question concerned their interactive association with the outcome, both predictors were retained in the same model. To facilitate interpretation and reduce non-essential multicollinearity with the interaction term, predictors were mean centered prior to analysis.

###### 2.3.6.1.1 Group comparisons in habituation patterns

To directly compare habituation patterns between treatment-referred youth and a control group without a history of antisocial behavior, we conducted additional linear mixed-effects analyses separately for the left and right amygdala ROIs. For these analyses, time (early, late) and condition (happy, neutral, angry, fearful, sad) were included as within-subject factors and group (treatment-referred young adults, controls) as a between-subject factor. Winsorized ROI beta estimates were analyzed using linear mixed-effects models including fixed effects for group, time, condition, and all lower- and higher-order interactions, with a random intercept for participant and, where supported by the data, a random slope for time. Follow-up analyses examined Late–Early change scores (Δβ = late − early) within each condition and compared these between groups using estimated marginal means with Bonferroni correction for multiple comparisons.

##### 2.3.6.2 The additive effect of amygdala habituation on recidivism

To test the predictive effect of amygdala habituation on recidivism, we modeled time-to-event data using four Cox proportional hazard regression models (separately for left and right amygdala, for general and serious recidivism). The primary outcome of these models was 1) time to general recidivism (in days) and 2) time to serious recidivism (in days).

###### 2.3.6.2.1 Predictors and variable selection

For all four models (left and right amygdala, general and serious recidivism), the same candidate predictors were used as in the ROI models, including Internalizing and Externalizing problems, ACE total score, Age at baseline, Prior Delinquency, Ethnicity and Cannabis use. Moreover, we now added amygdala habituation (difference scores in activation between Late vs. Early) as a predictor. Similar to our ROI models, we explicitly modeled the interaction between Internalizing and Externalizing problems. Categorical variables were dummy-coded. To account for missing values in the predictors, we used multiple imputations (m = 10) using the *mice* package under the MAR assumption. Note that the time-to-event (time to recidivism) and event indicator (recidivism: yes/no) were not imputed, given that censoring already accounts for incomplete follow-up data. To account for sampling variability and uncertainty from missing data, parameter estimates were pooled using Rubin's rules.

To perform robust variable selection for our models, we used a penalized Cox model (glmnet) with repeated 10-fold cross-validation (25 repeats). Within each imputed dataset and repeat, a term was considered “selected” if any associated coefficient (including factor dummies) was non-zero; we then aggregated to a stability-selection frequency per term across all imputations and repeats. To limit overfitting, we applied an events-per-variable (EPV) cap (target ≈10); if the selected set exceeded ⌊events/10⌋, we retained the most stable predictors. Prespecified variables could be forced in (penalty factor = 0) on a theoretical basis. Continuous predictors were standardized during penalization to ensure comparability, but final models were refit on the original scale without penalization to yield unbiased, interpretable hazard ratios (HRs). Hazard ratios (HRs) with 95% confidence intervals were pooled across imputations using Rubin's rules.

###### 2.3.6.2.2 Model selection and evaluation

We tested whether amygdala habituation improved prediction beyond the consensus model using a pooled Wald test and changes in performance metrics. If amygdala habituation was not selected by penalization, we ran a sensitivity analysis by adding it manually.

To assess model performance, we first evaluated discrimination using Harrell's C-index (overall concordance) and time-dependent ROC/AUC at pre-specified follow-up times (6, 12, 18, 24, and 30 months) using the risksetROC package in R. Overall accuracy was assessed using the Brier score and the integrated Brier score (IBS) over the time horizon. To correct for optimistic performance estimates, we used 0.632 bootstrap (B = 100) using the *pec* package in R. Calibration was examined at the same time points using pseudo-observations and non-parametric smoothing (method = “nne”, q = 10) using the calPlot function from the *pec* package in R.

##### 2.3.6.3 Assumption checks

The proportional hazards (PH) assumption was tested using the scaled Schoenfeld residuals. For both general and serious recidivism, the PH assumption was violated for the interaction between Internalizing and Externalizing problems, which was resolved by including this interaction-term as a time-varying predictor in the model. None of the models showed evidence of influential outliers (through inspection of deviance residuals) or nonlinearity in continuous predictors (through inspection of Martingale residual plots). Calibration plots and Brier scores (at 6, 12, 18, 24, and 30 months) (Zijlmans et al., 2021a, Zijlmans et al., 2021b) indicated good predictive accuracy across time points. To account for multiple comparisons, while also accounting for the high correlation between general and serious recidivism (r = 0.672), we applied a modified Bonferroni correction adjusted for correlated variables (Sankoh et al., 1997) of α = .05/1.98 = 0.025.

##### 2.3.6.4 Bias mitigation and reproducibility

Several measures were implemented to reduce potential sources of bias and improve reproducibility. Participant inclusion and exclusion criteria were defined a priori and applied consistently across groups. With regard to experimental conduct, all participants completed the same standardized task protocol and MRI acquisition procedure. Psychological symptoms were assessed using standardized instruments, and recidivism was assessed using prospectively collected official records, reducing reliance on subjective outcome assessment. Participants were excluded from particular analyses only if they did not have important information available (e.g. because controls had no data on internalizing and externalizing symptoms, they were excluded from the main hypothesis tests, or because not all participants completed the fMRI task) or if inclusion would have negatively influenced the robustness, reliability and validity of the results (e.g. participants with motion above the threshold were removed). MRI acquisition, preprocessing, and ROI definitions followed standardized procedures implemented in SPM12 and MarsBaR. Model specifications, covariates, and habituation contrasts were predefined based on prior literature and study hypotheses. Sensitivity analyses (e.g. winsorization to prevent influence of outliers), were conducted to assess robustness of findings. Whole-brain analyses were reported separately from hypothesis-driven ROI analyses to distinguish exploratory from confirmatory analyses. Finally, across the manuscript, habituation indices were prioritized because prior work suggests they may show stronger test–retest reliability than mean activation estimates.

### 2.4 Results

#### **2.4.1** **Whole brain results in** treatment-referred **young adults and controls**

Whole brain analyses (see Supplementary Table 1) indicated that the contrast Face > Control yielded significantly more activation in the Right Inferior Occipital Gyrus, just upstream of the fusiform face area, and the Right Precentral Gyrus. Finally, the Contrast Fearful Late > Fearful Early revealed more activity in the Right Insula, signaling increased sensitization to fearful faces across the whole sample (n = 122). Note that these whole brain analyses were not dependent on cannabis use (see Supplementary materials table 18).

#### **2.4.2** **Task performance in** treatment-referred **young adults and controls**

##### 2.4.2.1 Accuracy

To assess task performance, we first performed a repeated measures analysis of variance (ANOVA) with Condition (Happy, Neutral, Angry, Sad, Fearful, Control) and Group (Treatment-referred (n = 98) vs. Control (n = 24)) on mean accuracy (proportion correct responses). This analysis revealed a significant main effect of condition, *F*(1, 600) = 3.41, *p* = .005, ηp^2^ = 0.03. Across conditions, accuracy was highest in the Control Condition (M = 0.984, SD = 0.114) and lowest in Neutral Face Condition (M = 0.945, SD = 0.108). However, follow-up tests did not reveal any significant pairwise comparisons between conditions, all p's > 0.128. In addition, there was a main effect of group, *F*(1, 200) = 3.41, *p* = .005, ηp^2^ = 0.05, indicating that healthy controls showed higher accuracy (M = 0.981, SD = 0.020) on the task than treatment-referred young adults (M = 0.937, SD = 0.086), with a difference of 4.4%, 95% CI [0.9, 7.9], *p* = .014. The group × condition interaction was not significant, *F*(1, 600) = 0.94, *p* = .45.

##### 2.4.2.2 Reaction times

Next, we performed an ANOVA with Condition and Group on mean reaction times. The analysis revealed a significant main effect of condition, *F*(5, 595) = 580.35, *p* < .001, η_p_^2^ = 0.830, and group, *F*(1, 119) = 12.66, *p* < .001, η_p_^2^ = 0.096, which was qualified by a significant interaction of condition and group, *F*(5, 595) = 4.58, *p* < .001, η_p_^2^ = 0.037. Overall, individuals from the treatment-referred young adult group (M = 2.852, SD = 0.251 s, n = 98) were slower to respond than the control group (M = 2.661, SD = 0.184 s, n = 24), with a difference of −0.166, 95% CI [−0.259, −0.074], *p* < .001. Group differences were observed across all face conditions, but not for the control condition, *p* = .155. Reaction time differences were most pronounced for Neutral faces, ΔM = −0.292, 95% CI [−0.360, −0.049], *p* < .001, followed by Angry faces, ΔM = −0.204, 95% CI [−0.360, −0.049], *p* = .010, followed by Fearful faces, ΔM = −0.190, 95% CI [−0.360, −0.049], *p* < .001, followed by Happy faces, ΔM = −0.162, 95% CI [−0.360, −0.049], *p* = .015, followed by Sad faces, ΔM = −0.144, 95% CI [−0.360, −0.049], *p* = .009. Across conditions, Reaction times were fastest in Happy face condition (M = 2.508, SD = 0.293) and slowest in the control condition (M = 3.957, SD = 0.015).

### 2.5 The effect of internalizing and externalizing problems on amygdala habituation within treatment-referred young adults

#### 2.5.1 Left amygdala

Within the treatment-referred group, a linear mixed-effects model was fitted to examine whether interacting levels of internalizing and externalizing problems were associated with left amygdala habituation, and whether this effect varied by emotional face condition. The baseline model included fixed effects for time, condition, their interaction, all covariates, and random intercepts and slopes for time. Overall, for the left amygdala, 56 participants showed neural amygdala habituation, 42 participants showed neural sensitization, and no participants showed no change. Exploratory subgroup comparisons between participants showing habituation versus sensitization within each ROI are presented in the supplementary materials (Tables S12–13).

Adding the Internalizing × Externalizing × Time interaction to the baseline model did not significantly improve model fit, χ^2^(1) = 4.887, p = .087, ΔAIC = −0.887, ΔR^2^_m_ = 0.014, ΔR^2^꜀ = −0.001, nor did adding the Internalizing × Externalizing × Time × Condition term, χ^2^(4) = 7.75, *p* = .458, suggesting no interactive effect of Internalizing and Externalizing and providing no evidence for condition-specific differences. For full transparency, the full fixed-effects estimates are provided in the supplementary materials but are not interpreted further in the main text.

Next, we examined task effects independent of the interaction between Internalizing and Externalizing in the baseline model. Averaged across conditions, Late vs Early was higher (Δβ = 2.894, SE = 2.751, 95% CI [−2.571, 8.358], p = .296), although not significantly. The main effect of Condition, *F*(4, 736.09) = 1.69, p = .151 and the Time × condition interaction were not significant, *F*(4, 736.09) = 2.17, p = .070.

**Fig. 2:**
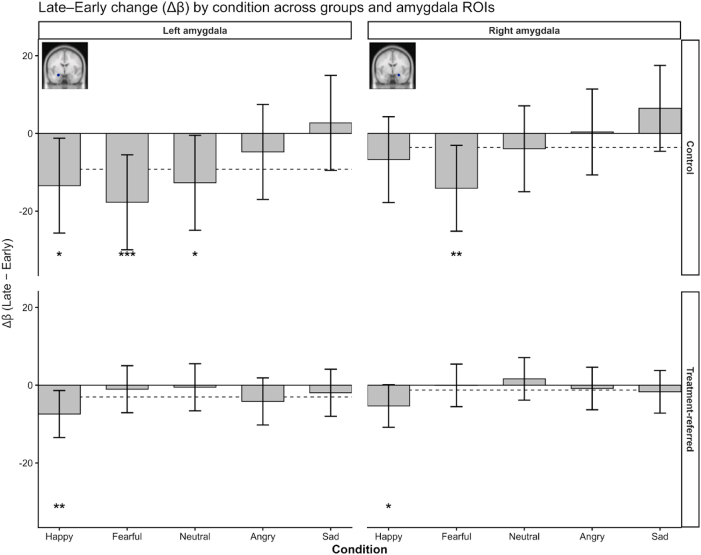
Left and right figures show the Late–Early change (Δβ) per Face Condition with 95% CIs, with the dashed line indicating the grand-mean Δβ across conditions in treatment-referred young adults (lower panels) and controls (upper panels). Stars mark Holm-adjusted significance for the within-condition Late–Early contrast (p < .05 = *, p < .01 = **, p < .001 = ***). Habituation was significantly stronger for the happy condition relative to the overall observed pattern in the treatment-referred individuals for both the left and right amygdala. For the control group, habituation was significantly stronger for the fearful condition relative to the overall observed pattern for both the left and right amygdala. For the left amygdala, habituation was also stronger for happy and neutral faces in the control group.

The baseline analysis did reveal significant fixed effects. First, there was a significant fixed effect of Habituation type, b = −4.21, SE = 1.309, t(91) = −3.22, p = .002, 95% CI [−6.776, −1.644], suggesting that people who showed habituation, had lower overall left amygdala parameter estimate scores than the grand mean across habituation types. In addition, the Time × Happy deviation contrast was significant, b = 2.348, SE = 0.923, t(728) = 2.54, p = .011, 95% CI [0.539, 4.157]), indicating that the Late vs. Early change for Happy was more negative than the average across conditions (deviation ΔΔ*β* = −4.70, *p* = .011, 95% CI [−8.31,−1.08]). This reflects stronger habituation for the happy condition relative to the overall observed pattern (see Fig. 2). The fixed effect for the Happy condition was initially significant, b = −1.881, SE = 0.923, t(728) = −2.04, p = .042, 95% CI [−3.69, −0.072], indicating that in the happy condition, participants showed lower overall left amygdala parameter estimate scores than the grand mean across conditions. However, this effect did not survive the Bonferroni correction adjusted for correlated variables. No other fixed effects were significant, all *p*'s > 0.093. Full fixed-effects estimates for the baseline model are provided in the supplementary materials.

#### 2.5.2 Right amygdala

Just as for the Left amygdala, a linear mixed-effects model was fitted to examine whether interacting levels of internalizing and externalizing problems predicted right amygdala habituation, and whether this effect varied by emotional face condition. The baseline model included fixed effects for time, condition, their interaction, all covariates, and random intercepts and slopes for time. For the right amygdala, 47 participants showed neural amygdala habituation, 50 participants showed neural sensitization, and 1 participant showed no change. Given that the “no-change” group would be too small for meaningful statistical analyses based on Habituation type, data for this individual was excluded from the linear mixed model.

Adding the Internalizing × Externalizing × Time interaction did not significantly improve model fit, χ^2^(1) = 0.306, p = .86, ΔAIC = 3.694, ΔR^2^_m_ = 0.001, ΔR^2^꜀ = −0.001, nor did adding the Internalizing × Externalizing × Time × Condition term, χ^2^(4) = 6.487, p = .59, suggesting no interactive effect of Internalizing and Externalizing and providing no evidence for condition-specific differences. For full transparency, the full fixed-effects estimates are provided in the supplementary materials but are not interpreted further in the main text. In the baseline model, averaged across conditions, Late vs Early was higher, Δβ = 1.51, SE = 2.522, 95% CI [−3.499, 6.518], p = .551), although not significantly. Omnibus tests further failed to reveal a main effect of condition: *F*(4, 736.09) = 2.18, p = .068 or a Time × condition interaction, *F*(4, 736.09) = 2.01, p = .090.

The baseline analysis revealed several significant fixed effects. First, there was a significant fixed effect for the Happy condition (b = −2.342, SE = 0.884, *t*(728) = −2.649, *p* = .008, 95% CI [−4.074, −0.60]), indicating that in the happy condition, treatment-referred participants showed lower overall right amygdala parameter estimate scores than the grand mean across conditions. Moreover, the Time × Happy deviation contrast was significant, (b = 2.356, SE = 0.884, *t*(728) = 2.665, *p* = .008, 95% CI [0.623, 4.088]), indicating that the Late vs. Early change for Happy was more negative than the average across conditions (deviation ΔΔ*β* = −4.71, *p* = .008, 95% CI [−8.176, −1.246], signifying stronger habituation for the happy condition relative to the overall observed pattern (see Fig. 2). No other fixed effects were significant, all *p*'s > 0.067. Full fixed-effects estimates for the baseline model are provided in the supplementary materials.

### 2.6 Contextualizing habituation patterns in treatment-referred youth relative to controls

We next examined whether treatment-referred youth with a history of antisocial behavior showed different habituation patterns compared to controls, to contextualize the previous findings. In these additional analyses, we tested whether habituation differed between treatment-referred young adults and controls by examining Group x Time and Group x Time x Condition effects.

#### 2.6.1 Left amygdala

For the left amygdala, there was no significant interaction between Group and Time, *F*(1, 122) = 1.103, *p* = . 296. However, there was a significant interaction effect between Group, Time and Condition, *F*(4, 976) = 4.086, *p* = .003. Follow-up condition-specific contrasts showed that controls had more negative Late-Early change scores than the treatment-referred group for fearful faces (Δβ = −16.703, SE = 6.956, 95% CI [−30.336, −3.07], *p* = .016), consistent with stronger habituation in controls and reduced habituation in the treatment-referred group for these conditions.

#### 2.6.2 Right amygdala

For the right amygdala, a similar pattern emerged (see supplementary materials for the full regression tables). There was no significant interaction between Group and Time, *F*(1, 122) = 0.198, *p* = .657. However, there was a significant interaction effect between Group, Time and Condition, *F*(4, 976) = 4.182, *p* < .002. Follow-up condition-specific contrasts again showed that controls had more negative Late-Early change scores than the treatment-referred group for fearful faces (Δβ = −14.070, SE = 6.29, 95% CI [−26.399, −1.741], *p* = .025), consistent with reduced habituation in the treatment-referred group compared to controls.

### 2.7 The additive effect of internalizing and externalizing problems and amygdala habituation on recidivism within treatment-referred young adults

#### 2.7.1 Left amygdala, general recidivism

Next, we examined the association between left amygdala habituation, Internalizing x Externalizing, and time to general recidivism. The final Cox proportional hazards model included the interaction between internalizing and externalizing problems and left amygdala habituation (difference score late vs. early), but no other covariates were selected to be included. There was a significant interaction between Internalizing and Externalizing on general time to recidivism, HR = 1.84, 95% CI [1.05, 3.21], *p* = .032, indicating that interacting levels of internalizing and externalizing problems increased the risk of recidivism. The interaction did not show a time-varying effect, HR = 0.98 (anchor point at 6 months), 95% CI [0.80, 1.01], *p* = .082. The model showed reasonable discrimination, with Harrell's C = 0.518, AUC at 12 months = 0.510, and Integrated Brier Score (IBS) = 0.197. This is likely due to a limited number of events (n = 53), which results in a relatively low number of estimated events per variable (EPV).

The added value tests indicated that adding the interaction between Internalizing and Externalizing did significantly improve model fit, D_2_ test, *p* < .001, even after correcting for multiple comparisons using a Sankoh-Bonferroni adjusted alpha (α = .025). However, adding the interaction between Internalizing, Externalizing and left Amygdala habituation did not further improve model fit, D_2_ test, *p* = 1.00.

#### 2.7.2 Left amygdala, serious recidivism

Subsequently, we examined the association between left amygdala habituation, Internalizing x Externalizing, and time to serious recidivism. Again, the final Cox proportional hazards model included the interaction between internalizing and externalizing problems and left amygdala habituation (difference score late vs. early), but no other covariates were selected to be included. There was a significant interaction between Internalizing and Externalizing on time to serious recidivism, HR = 2.22, 95% CI [1.13, 4.35], *p* = .020. The interaction did not show a time-varying effect, HR = 0.87 (anchor point at 6 months), 95% CI [0.75, 1.00], *p* = .056. The model showed reasonable discrimination, with Harrell's C = 0.584, AUC at 12 months = 0.536, and Integrated Brier Score (IBS) = 0.151. This is likely due to a limited number of events (n = 31), which results in a relatively low number of estimated events per variable (EPV).

The added value tests indicated that adding the interaction between Internalizing and Externalizing did significantly improve model fit, D_2_ test, *p* < .001, even after correcting for multiple comparisons using a Sankoh-Bonferroni adjusted alpha (α = .025). However, adding the interaction between Internalizing, Externalizing and left Amygdala habituation did not further improve model fit, D_2_ test, *p* = .740.

#### 2.7.3 Right amygdala, general recidivism

Next, we examined the association between right amygdala habituation, Internalizing x Externalizing, and time to general recidivism. The final Cox proportional hazards model included the interaction between internalizing and externalizing problems and right amygdala habituation (difference score late vs. early), but no other covariates were selected to be included. There was a significant interaction between Internalizing and Externalizing on general time to recidivism, HR = 1.85, 95% CI [1.06, 3.20], *p* = .029, indicating that interacting levels of internalizing and externalizing problems increased the risk of recidivism. The interaction did not show a time-varying effect, HR = 0.90 (anchor point at 6 months), 95% CI [0.80, 1.01], *p* = .071. The model showed reasonable discrimination, with Harrell's C = 0.518, AUC at 12 months = 0.457, and Integrated Brier Score (IBS) = 0.199. This is likely due to a limited number of events (n = 53), which results in a relatively low number of estimated events per variable (EPV).

The added value tests indicated that adding the interaction between Internalizing and Externalizing did significantly improve model fit, D_2_ test, *p* = .001, even after correcting for multiple comparisons using a Sankoh-Bonferroni adjusted alpha (α = .025). However, adding the interaction between Internalizing, Externalizing and right Amygdala habituation did not further improve model fit, D_2_ test, *p* = .999.

#### 2.7.4 Right amygdala, serious recidivism

Finally, we examined the association between right amygdala habituation, Internalizing x Externalizing, and time to serious recidivism. Again, the final Cox proportional hazards model included the interaction between internalizing and externalizing problems and right amygdala habituation (difference score late vs. early), but no other covariates were selected to be included. There was a significant interaction between Internalizing and Externalizing on time to serious recidivism, HR = 2.17, 95% CI [1.12, 4.19], *p* = .021, indicating that interacting levels of internalizing and externalizing problems increased the risk of serious recidivism. The interaction did not show a time-varying effect, HR = 0.87 (anchor point at 6 months), 95% CI [0.75, 1.01], p = .059. The model showed reasonable discrimination, with Harrell's C = 0.586, AUC at 12 months = 0.586, and Integrated Brier Score (IBS) = 0.149. This is likely due to a limited number of events (n = 31), which results in a relatively low number of estimated events per variable (EPV).

The added value tests indicated that adding the interaction between Internalizing and Externalizing did significantly improve model fit, D_2_ test, *p* < .001, even after correcting for multiple comparisons using a Sankoh-Bonferroni adjusted alpha (α = .025). However, adding the interaction between Internalizing, Externalizing and right Amygdala habituation did not further improve model fit, D_2_ test, *p* = .852.

### 2.8 General discussion

Slower amygdala habituation to emotional faces may reflect heightened emotional reactivity and reduced engagement with social cues (Bas-Hoogendam et al., 2019; Bilek et al., 2019; Holz et al., 2021; Kleinhans et al., 2009, 2016), processes that may interfere with learning from others’ signals and therefore increase risk for persistent aggression (Jansen, 2022; Nikolic et al., 2022; van Dongen et al., 2024). Prior studies suggest that the impact of such neural patterns may depend on the presence of co-occurring internalizing and externalizing problems, which could either amplify risk for persistent antisocial behavior or, in some cases, buffer against it (Shi et al., 2020; Berhe et al., 2023; Beauchaine and Thayer, 2015; Schettini et al., 2019). In the current study, we tested whether the combination of internalizing and externalizing problems was related to amygdala habituation to emotional faces in treatment-referred young adults with a history of antisocial behavior. We also examined whether this combination, together with amygdala habituation, predicted criminal recidivism. Additionally, behavioral, ROI and whole brain analyses were conducted within the treatment-referred and a control group provided insight into broader task and habituation contextualization and validity.

We found no evidence for our first hypothesis that the combination of internalizing and externalizing problems was related to slower amygdala habituation to emotional faces in treatment-referred young adults with a history of antisocial behavior, nor for emotion-specific differences in this effect. This might be explained in several ways. First, while there is always the possibility of a true absence of an association, we observed a range restriction in internalizing and externalizing problem scores in the treatment-referred young adult group, with most of them showing high scores on both scales. Potentially, this range restriction in internalizing and externalizing may have attenuated the potential associations with left and right amygdala habituation. In line with this idea, scatterplots of habituation against internalizing and externalizing scores showed flat relationships (see Fig. 2, indicating near-zero correlations. Second, for almost all face conditions (except the Happy condition for left amygdala), we observed relatively large standard errors and correspondingly wide confidence intervals, indicating high between-subject variability and reduced precision in estimating mean habituation slopes. This suggests that although the overall habituation pattern was robust at the group level, the noisiness of individual differences may have limited our ability to detect associations with internalizing and externalizing problems (Elliott et al., 2020; Fröhner et al., 2019; Hedge et al., 2018; Pezzoli et al., 2023; Varkevisser et al., 2024). Future studies may increase sensitivity to detect association between amygdala habituation and internalizing and externalizing problems by increasing the number of trials per condition and/or through adaptive design optimization (Pezzoli et al., 2023). A starting point to optimize the design and elicit more individual variation could be to make the face processing task more difficult (following the observation that treatment-referred young adults showed high task performance, both in accuracy and reaction times) or to introduce stronger social context manipulations signaling threat or distress (van de Groep et al., 2023). Other potential strategies to increase sensitivity to detect effects include expanding the range of internalizing and externalizing problems across the lower end of the spectrum, or modeling the associations across more fine-grained time courses (Flechsenhar et al., 2022; Pezzoli et al., 2023; van de Groep et al., 2023). Moreover, future studies could also examine more specific dimensions than externalizing behaviors, such as psychopathic traits, callous-unemotional traits, impulsivity, or aggression, which may show distinct associations with amygdala habituation to social and emotional cues.

In our study, we found no support for differential habituation to negatively or neutrally valenced faces in treatment-referred young adults, as we initially expected based on prior fMRI studies using emotional face processing tasks looking at mean-level amplitude (Jeung-Maarse et al., 2023; McCloskey et al., 2016; Coccaro et al., 2007; Dotterer et al., 2017; Jones et al., 2009; Sebastian et al., 2021; Berluti et al., 2023). Because our models included both random intercepts and slopes for time, we could separate mean-level amygdala activation from habituation effects, strengthening the conclusion that negative and neutral faces showed similar change over time within the treatment-referred group, rather than showing selective patterns for threatening and ambiguous negative cues (e.g. angry and neutral faces) (Jeung-Maarse et al., 2023; McCloskey et al., 2016; Coccaro et al., 2007; Hardi et al., 2025) or distress cues in others (e.g. fearful and sad faces) (Zhao et al., 2025). Importantly, however, our analyses did reveal stronger habituation to happy faces, suggesting that affiliative social cues may be processed differently in the treatment-referred young adults compared to other emotional facial expressions. More specifically, our finding of stronger habituation for the happy condition compared to the overall observed pattern across conditions may suggest a selective tendency to disengage from affiliative cues (Waller and Wagner, 2019). This may indicate that such signals are quickly appraised as non-threatening, leading to more disengagement of salience-related neural systems (Hardi et al., 2025). Alternatively, it could also reflect reduced motivation to engage with affiliative cues, which could potentially undermine learning from positive social interactions (Waller and Wagner, 2019). Future research should examine these possibilities in more detail, preferably by examining initial reactivity, mean-level responses and habituation trajectories, through application of more fine-grained time-course analyses (e.g. trial-to-trial level) (Flechsenhar et al., 2022; Pezzoli et al., 2023). Moreover, adaptive design optimization focusing on personalized optimization of social information salience (Pezzoli et al., 2023; Varkevisser et al., 2024), or stronger social context manipulations signaling threat or distress may clarify whether different patterns may emerge across different social contexts and task manipulations.

**Fig. 3:**
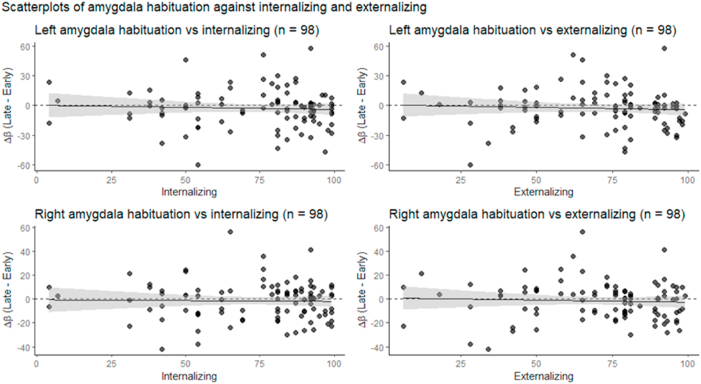
Scatterplots of amygdala habituation (Late–Early) against internalizing and externalizing scores across hemispheres and conditions; relationships are flat, consistent with near-zero correlations.

Additional analyses comparing the treatment-referred youth to gender-matched controls indicated that the control group was quicker to habituate to fearful faces than the treatment-referred group (see Fig. 3), with treatment-referred youth showing sustained activity throughout the task. Whereas previous studies have mostly indicated that youth with antisocial behavior show reduced mean amygdala responses to fearful faces (Dotterer et al., 2017; Jones et al., 2009; Sebastian et al., 2021; Berluti et al., 2023), the current study extends these results by showing that that the temporal dynamics and updating of socio-emotional processing may also be altered. Given that treatment-referred youth’ change scores between early and late blocks for fearful faces were close to zero (see Fig. 3), this finding fits more closely with a sustained threat or failure to update interpretation (Hardi et al., 2025), than a disengagement interpretation (Zhao et al., 2025).

#### 2.8.1 Interacting internalizing and externalizing problems are associated with increased recidivism hazard

Our findings further suggest that co-occurring internalizing symptoms appear to compound externalizing-related risk for recidivism. In general, this fits with literature suggesting that this co-occurrence may reflect more severe and persistent psychopathology and hence serve as a risk factor for negative adaptive functioning (Shi et al., 2020; Berhe et al., 2023) rather than as a protective factor (Beauchaine and Thayer, 2015; Schettini et al., 2019). This finding fits with a prior meta-analysis suggesting that combinations of internalizing and externalizing disorders show a small to moderate positive association with recidivism in youth aged 5-19 (Wibbelink et al., 2017), but extends these findings by demonstrating that this pattern also extends into early adulthood. It should be noted that although the cox Regression models revealed a significant statistical association between Internalizing and Externalizing on recidivism, the predictive value of all models was low. Although this is not uncommon in clinical samples and likely reflects heterogeneity, this poor discrimination again limits the clinical actionability of the current findings. Although our models already included multimodal predictors (e.g., brain measures, clinical symptom scores), enhancing prediction may require models that combine longitudinal information on symptom change with higher-resolution multimodal features, to better capture individual variability in risk.

**Fig. 4:**
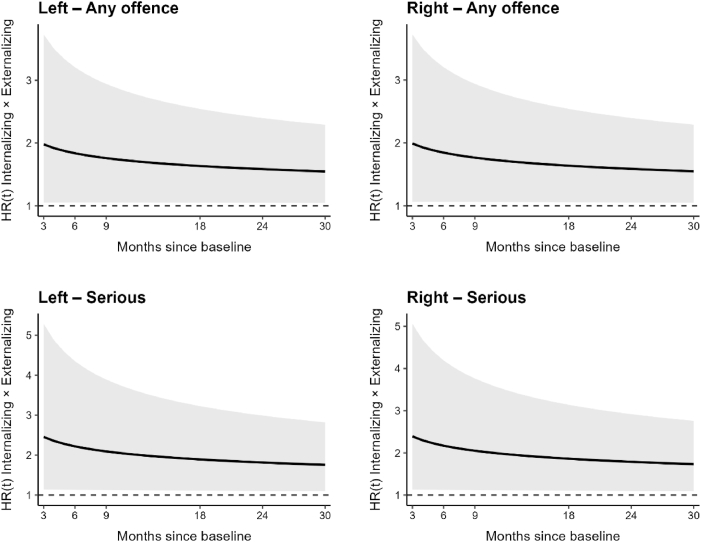
Hazard Ratios for the interaction between Internalizing and Externalizing for general recidivism (top) and serious recidivism (bottom), for the models including the left amygdala (left) and right amygdala (right).

Apart from the observed interaction effect between internalizing and externalizing on recidivism, visualization of the time-varying interaction between internalizing and externalizing on general and severe recidivism (see Fig. 4) revealed a trend of elevated early risk of recidivism that diminished over time. Although this pattern did not reach statistical significance and thus should be interpreted with caution, it is worth noting that the observed trend is consistent with psychological and criminological theories that indicate that co-occurring internalizing problems may drive early risk of recidivism by worsening existing vulnerabilities related to externalizing problems (Sampson and Laub, 1997), but reduce later risk through disengagement or withdrawal associated with increased maturation and anxiety (Bersani and Doherty, 2018; Monahan et al., 2009, 2013).

Finally, although our findings suggest that co-occurring internalizing symptoms appear to increase externalizing-related risk for recidivism, we found no evidence for our hypothesis that amygdala habituation contributes to or moderates this pattern. Again, the observed noisiness of individual differences in amygdala habituation, indicative of limited reliability compared to group-level indices, may have contributed to this lack of an effect (Elliott et al., 2020; Fröhner et al., 2019; Pezzoli et al., 2023; Varkevisser et al., 2024). Future studies should address this issue by developing and validating emotional face-processing tasks optimized to detect clinically meaningful individual differences (Elliott et al., 2020; Fröhner et al., 2019; Pezzoli et al., 2023; Varkevisser et al., 2024).

#### 2.8.2 Strengths and limitations

A key strength of the current study is our focus on neural habituation, rather than merely mean level amplitude, which provides more insight into potential temporal dynamics of social emotional processing. Moreover, we attempted to link neural habituation patterns to both dimensional indicators of psychopathology and recidivism, extending earlier work that hypothesized an association with social and adaptive functioning (Bas-Hoogendam et al., 2019; Bilek et al., 2019; Holz et al., 2021; Kleinhans et al., 2009, 2016), but did not test how amygdala habituation relates real-life functioning outside of experimental contexts (but see Berhe et al., 2023 for an exception). In addition, our dimensional approach focusing on interactions between internalizing and externalizing problems aligns well with contemporary dimensional frameworks such as The Hierarchical Taxonomy of Psychopathology (HiTOP) and Research Domain Criteria (RDOC) (Beauchaine and Hinshaw, 2020; Conradt et al., 2021; Insel et al., 2010; Kotov et al., 2021), recognizing limitations with more traditional classifications of psychopathology (e.g. DSM).

However, our study also has several limitations. Although our sample was relatively large for clinical neuroimaging standards, power to detect robust brain-behavior associations that involve complex interactions between internalizing and externalizing problems may have been limited (Elliott et al., 2020; Fröhner et al., 2019; Pezzoli et al., 2023; Varkevisser et al., 2024). Second, the shape-matching control condition may also have influenced task effects. Because some geometric shapes, such as rectangles and triangles, differ more strongly from faces than rounded shapes, the control condition may have differed from the face conditions in visual complexity and task difficulty, potentially affecting face > control contrasts and behavioral performance. At the same time, the use of more distinct shapes may have made the control task more engaging and reduced motivational differences between face and control trial. Likewise, the number of offense events observed within the sample may have limited the discrimination ability of the cox regression models. Moreover, although our habituation index matches with current practices in the scientific literature, operationalization as merely an Early–Late difference, may have limited our ability to capture nonlinear within-run trajectories or early-phase changes. Another limitation of the study is that it was only focused on male participants. Although antisocial behavior and recidivism are more commonly observed in males (Eme, 2020), future studies should examine whether the effects generalize to other genders. Finally, because the experimental and control groups differed in ethnic composition, it could be possible that group differences were introduced by how often individuals had to process in-group and out-group faces. Thus, between-group comparisons should be interpreted with caution, and future studies should use ethnically diverse and counterbalanced face stimuli or explicitly model the match between participant and stimulus ethnicity to disentangle group effects from potential in-group and out-group processing effects.

## 3 Conclusion

Using behavioral and fMRI methods, the current study examined patterns of amygdala habituation and their association with recidivism in treatment-referred young adults. Amygdala activation in treatment-referred young adults habituated stronger for happy faces compared to the overall pattern across different emotions, signaling that affiliative social cues may be rapidly appraised as non-threatening, leading to more disengagement of salience-related neural systems such as the amygdala. Moreover, while co-occurring internalizing symptoms appear to compound externalizing-related risk for recidivism, we found that amygdala habituation did not add predictive value for recidivism in the current sample Taken together, these findings suggest that amygdala habituation to emotional faces may help characterize emotion-specific patterns of social-emotional processing, but its value for explaining recidivism in treatment-referred young adults with antisocial histories remains limited.

## Declaration of generative AI and AI-assisted technologies in the writing process

During the preparation of this work the author(s) used chat-GPT to improve readability and language. After using this tool/service, the author(s) reviewed and edited the content as needed and take(s) full responsibility for the content of the published article.

## Funding

This research is supported by Convergence Healthy Start, a program of the Convergence Alliance – Delft University of Technology, Erasmus University Rotterdam and Erasmus Medical Center - to improve the future of new generations and by the De Verre Bergen Foundation. De Verre Bergen Foundation is a venture philanthropy organization that aims to build a better Rotterdam through substantial investments in innovative, impactful social ventures. The financer was not involved in the design of the study nor the drafting of the manuscript. Furthermore, the financer was not involved in the process of data collection, analysis, and interpretation.

## CRediT authorship contribution statement

**Ilse H. van de Groep:** Conceptualization, Data curation, Formal analysis, Investigation, Visualization, Writing – original draft, Writing – review & editing. **Josjan Zijlmans:** Conceptualization, Resources, Writing – review & editing. **Reshmi Marhe:** Conceptualization, Data curation, Methodology, Resources, Supervision, Validation, Writing – review & editing.

## Declaration of competing interest

The authors declare that they have no known competing financial interests or personal relationships that could have appeared to influence the work reported in this paper.

## Data Availability

Data will be made available on request.

## Footnotes

1 Note that running a mixed model using both Time and Face condition as random intercepts and slopes resulted in a singular fit. Therefore, we decided to only include random intercepts and slopes for Time.
