# Supplementary material for: Emotion-specific amygdala habituation in treatment-referred young adults with antisocial histories: links with internalizing and externalizing symptoms and future recidivism
Source: Neuroimage Rep. 2026 Jun 11;6(3):100371. doi: 10.1016/j.ynirp.2026.100371 (PMC13273875; doi:10.1016/j.ynirp.2026.100371)
Supplement: Multimedia component 1 [file mmc1.docx]

| Supplementary Table 1 |  | | | |  |  |
| --- | --- | --- | --- | --- | --- | --- |
| MNI coordinates of local maxima activated the contrast “Face vs. Control”, “Control vs. Face” and “Fearful Late vs. Fearful Early” for the Whole brain ANOVA for the Face Processing task. Results were cluster-corrected using p <0.001 as an initial threshold.  None of the other contrasts resulted in significant activation after FWE correction. | | | | | | |
| Area of activation | MNI Coordinates | | | Test statistic | | Cluster Size |
|  | x | y | z | *T* | |  |
| *Face vs. Control* |  |  |  |  | |  |
| Occipital_inf_R | 30 | -91 | 0 | 6.50 | | 94 |
| Precentral_R | 33 | 9 | 28 | 4.34 | | 106 |
| *Control vs. Face* |  |  |  |  | |  |
| Occipital_sup_L | -15 | -60 | 21 | 4.4 | | 66 |
| *Fearful Late vs. Fearful Early* |  |  |  |  | |  |
| Insula_R | 26 | 23 | 0 | 5.06 | | 90 |
|  |  |  |  |  | |  |

| Supplementary Table 2 | | | | | | |
| --- | --- | --- | --- | --- | --- | --- |
| Fixed effect table of the baseline model – Left amygdala | | | | | | |
| **Term** | **Estimate** | **Std. Error** | **df** | **t value** | **Pr(>\|t\|)** | **95% CI** |
| (Intercept) | -13.032 | 13.328 | 91.160 | -0.978 | .331 | [-39.154, 13.09] |
| Overall habituation effect across emotions | 1.447 | 1.368 | 91.001 | 1.058 | .293 | [-1.235, 4.128] |
| Happy | -1.881 | 0.923 | 728.000 | -2.038 | .042 | [-3.69, -0.072] |
| Neutral | 1.555 | 0.923 | 728.000 | 1.684 | .093 | [-0.254, 3.364] |
| Angry | 0.258 | 0.923 | 728.000 | 0.279 | .780 | [-1.551, 2.067] |
| Fearful | -0.756 | 0.923 | 728.000 | -0.819 | .413 | [-2.565, 1.053] |
| Internalizing problems | 0.310 | 1.826 | 90.999 | 0.170 | .865 | [-3.269, 3.889] |
| Externalizing problems | 0.032 | 1.838 | 90.999 | 0.018 | .986 | [-3.571, 3.635] |
| Habituation type | -4.210 | 1.309 | 90.999 | -3.216 | .002 | [-6.776, -1.644] |
| Delinquency | 0.776 | 1.358 | 90.999 | 0.572 | .569 | [-1.885, 3.437] |
| ACEs | -0.869 | 0.747 | 90.999 | -1.164 | .248 | [-2.332, 0.595] |
| Age | 0.774 | 0.619 | 90.999 | 1.250 | .215 | [-0.44, 1.987] |
| Ethnicity | 0.147 | 0.565 | 90.999 | 0.260 | .796 | [-0.961, 1.254] |
| Cannabis use | -0.352 | 0.388 | 90.999 | -0.909 | .366 | [-1.112, 0.407] |
| Happy-face habituation deviation | 2.348 | 0.923 | 728.000 | 2.544 | .011 | [0.539, 4.157] |
| Neutral-face habituation deviation | -1.295 | 0.923 | 728.000 | -1.403 | .161 | [-3.104, 0.514] |
| Angry-face habituation deviation | 0.702 | 0.923 | 728.000 | 0.760 | .447 | [-1.107, 2.511] |
| Fearful-face habituation deviation | -1.137 | 0.923 | 728.000 | -1.232 | .218 | [-2.946, 0.672] |

| Supplementary Table 3 | | | |
| --- | --- | --- | --- |
| Simple Late–Early contrasts per condition for the baseline model- Left amygdala | | | |
| **Condition** | **Δβ (Late−Early)** | **95% CI** | **p** |
| Happy | -7.589 | [-14.059, -1.12] | .021 |
| Neutral | -0.305 | [-6.774, 6.165] | .926 |
| Angry | -4.298 | [-10.767, 2.172] | .193 |
| Fearful | -0.620 | [-7.089, 5.849] | .851 |
| Sad | -1.658 | [-8.127, 4.811] | .615 |

| Supplementary Table 4 | | | |
| --- | --- | --- | --- |
| Time × Condition: slope deviations (vs grand mean) for the baseline model - Left amygdala | | | |
| **Condition** | **ΔΔβ (vs mean)** | **95% CI** | **p** |
| Happy | -4.696 | [-8.314, -1.078] | .011 |
| Neutral | 2.589 | [-1.029, 6.207] | .161 |
| Angry | -1.404 | [-5.022, 2.214] | .447 |
| Fearful | 2.274 | [-1.344, 5.892] | .218 |
| Sad | 1.236 | [-2.382, 4.854] | .503 |

| Supplementary Table 5 | | | | | | |
| --- | --- | --- | --- | --- | --- | --- |
| Fixed effect table for the baseline model – Right amygdala | | | | | | |
| **Term** | **Estimate** | **Std. Error** | **df** | **t value** | **Pr(>\|t\|)** | **95% CI** |
| (Intercept) | 4.929 | 12.575 | 90.198 | 0.392 | .696 | [-19.718, 29.575] |
| Overall habituation effect across emotions | 0.755 | 1.254 | 90.000 | 0.602 | .549 | [-1.703, 3.212] |
| Happy | -2.342 | 0.884 | 719.997 | -2.649 | .008 | [-4.074, -0.609] |
| Neutral | 0.563 | 0.884 | 719.997 | 0.637 | .524 | [-1.17, 2.295] |
| Angry | 1.584 | 0.884 | 719.997 | 1.792 | .074 | [-0.148, 3.317] |
| Fearful | 0.409 | 0.884 | 719.997 | 0.463 | .643 | [-1.323, 2.142] |
| Internalizing problems | 2.522 | 1.732 | 90.006 | 1.456 | .149 | [-0.874, 5.917] |
| Externalizing problems | -1.867 | 1.756 | 90.006 | -1.063 | .291 | [-5.309, 1.576] |
| Habituation type | 1.251 | 1.200 | 90.006 | 1.042 | .300 | [-1.101, 3.604] |
| Delinquency | 0.883 | 1.290 | 90.006 | 0.684 | .496 | [-1.646, 3.411] |
| ACEs | 0.483 | 0.706 | 90.006 | 0.684 | .495 | [-0.9, 1.866] |
| Age | -0.408 | 0.585 | 90.006 | -0.698 | .487 | [-1.553, 0.738] |
| Ethnicity | 0.563 | 0.537 | 90.006 | 1.048 | .297 | [-0.49, 1.617] |
| Cannabis use | -0.274 | 0.365 | 90.006 | -0.752 | .454 | [-0.99, 0.441] |
| Happy-face habituation deviation | 2.356 | 0.884 | 719.997 | 2.665 | .008 | [0.623, 4.088] |
| Neutral-face habituation deviation | -1.382 | 0.884 | 719.997 | -1.563 | .118 | [-3.114, 0.351] |
| Angry-face habituation deviation | -0.366 | 0.884 | 719.997 | -0.414 | .679 | [-2.099, 1.366] |
| Fearful-face habituation deviation | -0.511 | 0.884 | 719.997 | -0.578 | .563 | [-2.243, 1.222] |

| Supplementary Table 6 | | | |
| --- | --- | --- | --- |
| Simple Late–Early contrasts per condition for the baseline model- Right amygdala | | | |
| **Condition** | **Δβ (Late−Early)** | **95% CI** | **p** |
| Happy | -6.221 | [-12.234, -0.207] | .043 |
| Neutral | 1.254 | [-4.76, 7.267] | .683 |
| Angry | -0.777 | [-6.79, 5.237] | .800 |
| Fearful | -0.488 | [-6.501, 5.526] | .874 |
| Sad | -1.316 | [-7.329, 4.697] | .668 |

| Supplementary Table 7 | | | |
| --- | --- | --- | --- |
| Time × Condition: slope deviations (vs grand mean) for the baseline model - Right amygdala | | | |
| Happy | -4.711 | [-8.176, -1.246] | .008 |
| Neutral | 2.763 | [-0.702, 6.228] | .118 |
| Angry | 0.733 | [-2.732, 4.198] | .679 |
| Fearful | 1.022 | [-2.443, 4.487] | .563 |
| Sad | 0.193 | [-3.271, 3.658] | .913 |

| Supplementary Table 8 | | | | | | |
| --- | --- | --- | --- | --- | --- | --- |
| **Fixed effects for hierarchical Model 1 - Left amygdala** | | | | | | |
| **Term** | **Estimate** | **Std. Error** | **df** | **t value** | **Pr(>\|t\|)** | **95% CI** |
| (Intercept) | -3.604 | 13.637 | 91.197 | -0.264 | .792 | [-30.333, 23.124] |
| Overall habituation effect across emotions | 1.392 | 1.577 | 91.001 | 0.883 | .379 | [-1.698, 4.483] |
| Happy | -1.881 | 0.923 | 727.998 | -2.038 | .042 | [-3.69, -0.072] |
| Neutral | 1.555 | 0.923 | 727.998 | 1.684 | .093 | [-0.254, 3.364] |
| Angry | 0.258 | 0.923 | 727.998 | 0.279 | .780 | [-1.551, 2.067] |
| Fearful | -0.756 | 0.923 | 727.998 | -0.819 | .413 | [-2.565, 1.053] |
| Internalizing problems | -0.348 | 1.904 | 84.792 | -0.183 | .856 | [-4.08, 3.384] |
| Externalizing problems | -1.124 | 1.931 | 86.444 | -0.582 | .562 | [-4.908, 2.66] |
| Habituation type | -4.032 | 1.276 | 91.002 | -3.160 | .002 | [-6.533, -1.531] |
| Delinquency | 0.184 | 1.348 | 91.002 | 0.136 | .892 | [-2.459, 2.826] |
| ACEs | -0.855 | 0.727 | 91.002 | -1.176 | .243 | [-2.28, 0.57] |
| Age | 0.393 | 0.626 | 91.002 | 0.628 | .532 | [-0.834, 1.619] |
| Ethnicity | 0.108 | 0.550 | 91.002 | 0.197 | .844 | [-0.97, 1.187] |
| Cannabis use | -0.232 | 0.381 | 91.002 | -0.608 | .544 | [-0.979, 0.515] |
| Happy-face habituation deviation | 2.348 | 0.923 | 727.998 | 2.544 | .011 | [0.539, 4.157] |
| Neutral-face habituation deviation | -1.295 | 0.923 | 727.998 | -1.403 | .161 | [-3.104, 0.514] |
| Angry-face habituation deviation | 0.702 | 0.923 | 727.998 | 0.760 | .447 | [-1.107, 2.511] |
| Fearful-face habituation deviation | -1.137 | 0.923 | 727.998 | -1.232 | .218 | [-2.946, 0.672] |
| Overall habituation x Internalizing | 0.805 | 1.937 | 91.001 | 0.415 | .679 | [-2.991, 4.601] |
| Overall habituation x Externalizing | -0.212 | 1.895 | 91.001 | -0.112 | .911 | [-3.926, 3.502] |
| Internalizing x Externalizing | -2.564 | 1.210 | 84.182 | -2.118 | .037 | [-4.936, -0.192] |
| Time x Internalizing x Externalizing | 0.090 | 1.246 | 91.001 | 0.072 | .943 | [-2.352, 2.532] |

| Supplementary Table 9 | | | | | | |
| --- | --- | --- | --- | --- | --- | --- |
| **Fixed effects for hierarchical model 2 - Left amygdala** | | | | | | |
| **Term** | **Estimate** | **Std. Error** | **df** | **t value** | **Pr(>\|t\|)** | **95% CI** |
| (Intercept) | -3.604 | 13.638 | 91.193 | -0.264 | .792 | [-30.333, 23.125] |
| Overall habituation effect across emotions | 1.392 | 1.577 | 90.999 | 0.883 | .379 | [-1.698, 4.483] |
| Happy | -1.857 | 1.049 | 728.001 | -1.769 | .077 | [-3.914, 0.2] |
| Neutral | 1.656 | 1.049 | 728.001 | 1.578 | .115 | [-0.4, 3.713] |
| Angry | 0.223 | 1.049 | 728.001 | 0.212 | .832 | [-1.834, 2.28] |
| Fearful | -1.596 | 1.049 | 728.001 | -1.521 | .129 | [-3.653, 0.46] |
| Internalizing problems | -0.348 | 1.904 | 84.789 | -0.183 | .856 | [-4.08, 3.384] |
| Externalizing problems | -1.124 | 1.931 | 86.441 | -0.582 | .562 | [-4.908, 2.66] |
| Habituation type | -4.032 | 1.276 | 90.999 | -3.160 | .002 | [-6.533, -1.531] |
| Delinquency | 0.184 | 1.348 | 90.999 | 0.136 | .892 | [-2.459, 2.826] |
| ACEs | -0.855 | 0.727 | 90.999 | -1.176 | .243 | [-2.28, 0.57] |
| Age | 0.393 | 0.626 | 90.999 | 0.628 | .532 | [-0.834, 1.619] |
| Ethnicity | 0.108 | 0.550 | 90.999 | 0.197 | .844 | [-0.97, 1.187] |
| Cannabis use | -0.232 | 0.381 | 90.999 | -0.608 | .544 | [-0.979, 0.515] |
| Happy-face habituation deviation | 2.658 | 1.049 | 728.001 | 2.533 | .012 | [0.602, 4.715] |
| Neutral-face habituation deviation | -1.008 | 1.049 | 728.001 | -0.961 | .337 | [-3.065, 1.048] |
| Angry-face habituation deviation | 1.234 | 1.049 | 728.001 | 1.176 | .240 | [-0.823, 3.291] |
| Fearful-face habituation deviation | -1.327 | 1.049 | 728.001 | -1.264 | .207 | [-3.383, 0.73] |
| Overall habituation x Internalizing | 0.805 | 1.937 | 90.999 | 0.415 | .679 | [-2.991, 4.601] |
| Happy x Internalizing | -3.158 | 1.289 | 728.001 | -2.450 | .015 | [-5.684, -0.631] |
| Neutral x Internalizing | 2.328 | 1.289 | 728.001 | 1.806 | .071 | [-0.198, 4.855] |
| Angry x Internalizing | 0.555 | 1.289 | 728.001 | 0.431 | .667 | [-1.971, 3.082] |
| Fearful x Internalizing | 0.715 | 1.289 | 728.001 | 0.555 | .579 | [-1.811, 3.241] |
| Overall habituation x Externalizing | -0.212 | 1.895 | 90.999 | -0.112 | .911 | [-3.926, 3.502] |
| Happy x Externalizing | 2.316 | 1.261 | 728.001 | 1.837 | .067 | [-0.155, 4.788] |
| Neutral x Externalizing | -2.671 | 1.261 | 728.001 | -2.118 | .035 | [-5.143, -0.199] |
| Angry x Externalizing | 0.778 | 1.261 | 728.001 | 0.617 | .538 | [-1.694, 3.25] |
| Fearful x Externalizing | 0.120 | 1.261 | 728.001 | 0.096 | .924 | [-2.351, 2.592] |
| Internalizing x Externalizing | -2.564 | 1.210 | 84.179 | -2.118 | .037 | [-4.936, -0.192] |
| Happy-face habituation deviation x Internalizing | 1.347 | 1.289 | 728.001 | 1.045 | .296 | [-1.18, 3.873] |
| Neutral-face habituation deviation x Internalizing | -0.461 | 1.289 | 728.001 | -0.358 | .721 | [-2.987, 2.065] |
| Angry-face habituation deviation x Internalizing | 0.357 | 1.289 | 728.001 | 0.277 | .782 | [-2.169, 2.884] |
| Fearful-face habituation deviation x Internalizing | -0.958 | 1.289 | 728.001 | -0.743 | .458 | [-3.485, 1.568] |
| Happy-face habituation deviation x Externalizing | -1.020 | 1.261 | 728.001 | -0.809 | .419 | [-3.492, 1.452] |
| Neutral-face habituation deviation x Externalizing | -0.182 | 1.261 | 728.001 | -0.144 | .885 | [-2.654, 2.29] |
| Angry-face habituation deviation x Externalizing | -0.753 | 1.261 | 728.001 | -0.597 | .551 | [-3.225, 1.719] |
| Fearful-face habituation deviation x Externalizing | 1.467 | 1.261 | 728.001 | 1.163 | .245 | [-1.005, 3.939] |
| Overall habituation x Internalizing x Externalizing | 0.090 | 1.246 | 90.999 | 0.072 | .943 | [-2.353, 2.532] |
| Happy x Internalizing x Externalizing | -0.047 | 0.829 | 728.001 | -0.056 | .955 | [-1.672, 1.579] |
| Neutral x Internalizing x Externalizing | -0.159 | 0.829 | 728.001 | -0.192 | .848 | [-1.784, 1.467] |
| Angry x Internalizing x Externalizing | 0.061 | 0.829 | 728.001 | 0.074 | .941 | [-1.564, 1.687] |
| Fearful x Internalizing x Externalizing | 1.330 | 0.829 | 728.001 | 1.604 | .109 | [-0.295, 2.956] |
| Happy-face habituation deviation x Internalizing x Externalizing | -0.487 | 0.829 | 728.001 | -0.587 | .557 | [-2.113, 1.138] |
| Neutral-face habituation deviation x Internalizing x Externalizing | -0.455 | 0.829 | 728.001 | -0.549 | .583 | [-2.081, 1.17] |
| Angry-face habituation deviation x Internalizing x Externalizing | -0.841 | 0.829 | 728.001 | -1.015 | .311 | [-2.467, 0.784] |
| Fearful-face habituation deviation x Internalizing x Externalizing | 0.300 | 0.829 | 728.001 | 0.362 | .718 | [-1.325, 1.926] |
|  |  |  |  |  |  |  |

| Supplementary Table 10 | | | | | | |
| --- | --- | --- | --- | --- | --- | --- |
| **Fixed effects for hierarchical model 1 - Right amygdala** | | | | | | |
| **Term** | **Estimate** | **Std. Error** | **df** | **t value** | **Pr(>\|t\|)** | **95% CI** |
| (Intercept) | 7.398 | 13.166 | 90.223 | 0.562 | .576 | [-18.407, 33.204] |
| Overall habituation effect across emotions | 0.606 | 1.445 | 89.999 | 0.419 | .676 | [-2.226, 3.437] |
| Happy | -2.342 | 0.884 | 720.001 | -2.649 | .008 | [-4.074, -0.609] |
| Neutral | 0.563 | 0.884 | 720.001 | 0.637 | .524 | [-1.17, 2.295] |
| Angry | 1.584 | 0.884 | 720.001 | 1.792 | .074 | [-0.148, 3.317] |
| Fearful | 0.409 | 0.884 | 720.001 | 0.463 | .643 | [-1.323, 2.142] |
| Internalizing problems | 2.033 | 1.864 | 80.586 | 1.091 | .278 | [-1.619, 5.686] |
| Externalizing problems | -2.018 | 1.896 | 82.801 | -1.065 | .290 | [-5.734, 1.697] |
| Habituation type | 1.183 | 1.198 | 89.999 | 0.987 | .326 | [-1.166, 3.532] |
| Delinquency | 0.718 | 1.314 | 89.999 | 0.547 | .586 | [-1.856, 3.293] |
| ACEs | 0.490 | 0.704 | 89.999 | 0.695 | .489 | [-0.891, 1.87] |
| Age | -0.505 | 0.605 | 89.999 | -0.835 | .406 | [-1.69, 0.681] |
| Ethnicity | 0.553 | 0.537 | 89.999 | 1.029 | .306 | [-0.499, 1.605] |
| Cannabis use | -0.244 | 0.368 | 89.999 | -0.662 | .510 | [-0.965, 0.478] |
| Happy-face habituation deviation | 2.356 | 0.884 | 720.001 | 2.665 | .008 | [0.623, 4.088] |
| Neutral-face habituation deviation | -1.382 | 0.884 | 720.001 | -1.563 | .118 | [-3.114, 0.351] |
| Angry-face habituation deviation | -0.366 | 0.884 | 720.001 | -0.414 | .679 | [-2.099, 1.366] |
| Fearful-face habituation deviation | -0.511 | 0.884 | 720.001 | -0.578 | .563 | [-2.243, 1.222] |
| Overall habituation x Internalizing | 0.727 | 1.765 | 89.999 | 0.412 | .681 | [-2.733, 4.187] |
| Overall habituation x Externalizing | -0.333 | 1.727 | 89.999 | -0.193 | .847 | [-3.719, 3.052] |
| Internalizing x Externalizing | -0.765 | 1.181 | 79.622 | -0.648 | .519 | [-3.079, 1.55] |
| Overall habituation x Internalizing\ x Externalizing | 0.238 | 1.136 | 89.999 | 0.209 | .835 | [-1.989, 2.464] |

| Supplementary Table 11 | | | | | | |
| --- | --- | --- | --- | --- | --- | --- |
| **Fixed effects for hierarchical model 2 - Right amygdala** | | | | | | |
| **Term** | **Estimate** | **Std. Error** | **df** | **t value** | **Pr(>\|t\|)** | **95% CI** |
| (Intercept) | 7.398 | 13.166 | 90.228 | 0.562 | .576 | [-18.407, 33.203] |
| Overall habituation effect across emotions | 0.606 | 1.445 | 90.000 | 0.419 | .676 | [-2.226, 3.437] |
| Happy | -2.232 | 1.004 | 719.998 | -2.224 | .026 | [-4.199, -0.265] |
| Neutral | 0.576 | 1.004 | 719.998 | 0.574 | .566 | [-1.391, 2.543] |
| Angry | 1.830 | 1.004 | 719.998 | 1.823 | .069 | [-0.137, 3.797] |
| Fearful | -0.422 | 1.004 | 719.998 | -0.421 | .674 | [-2.39, 1.545] |
| Internalizing | 2.033 | 1.863 | 80.590 | 1.091 | .278 | [-1.619, 5.686] |
| Externalizing | -2.018 | 1.896 | 82.804 | -1.065 | .290 | [-5.734, 1.697] |
| Habituation type | 1.183 | 1.198 | 90.003 | 0.987 | .326 | [-1.166, 3.532] |
| Delinquency | 0.718 | 1.314 | 90.003 | 0.547 | .586 | [-1.856, 3.293] |
| ACEs | 0.490 | 0.704 | 90.003 | 0.695 | .489 | [-0.891, 1.87] |
| Age | -0.505 | 0.605 | 90.003 | -0.835 | .406 | [-1.69, 0.681] |
| Ethnicity | 0.553 | 0.537 | 90.003 | 1.029 | .306 | [-0.499, 1.605] |
| Cannabis use | -0.244 | 0.368 | 90.003 | -0.662 | .510 | [-0.965, 0.478] |
| Happy-face habituation deviation | 2.650 | 1.004 | 719.998 | 2.641 | .008 | [0.683, 4.617] |
| Neutral-face habituation deviation | -0.951 | 1.004 | 719.998 | -0.948 | .343 | [-2.918, 1.016] |
| Angry-face habituation deviation | -0.164 | 1.004 | 719.998 | -0.164 | .870 | [-2.131, 1.803] |
| Fearful-face habituation deviation | -0.810 | 1.004 | 719.998 | -0.807 | .420 | [-2.777, 1.157] |
| Overall habituation x Internalizing | 0.727 | 1.765 | 90.000 | 0.412 | .681 | [-2.733, 4.187] |
| Happy x Internalizing | -3.063 | 1.226 | 719.998 | -2.497 | .013 | [-5.466, -0.659] |
| Neutral x Internalizing | 1.772 | 1.226 | 719.998 | 1.445 | .149 | [-0.631, 4.176] |
| Angry x :Internalizing | -0.157 | 1.226 | 719.998 | -0.128 | .898 | [-2.56, 2.247] |
| Fearful x Internalizing | 1.971 | 1.226 | 719.998 | 1.608 | .108 | [-0.432, 4.375] |
| Overall habituation x Externalizing | -0.333 | 1.727 | 90.000 | -0.193 | .847 | [-3.719, 3.052] |
| Happy x Externalizing | 0.937 | 1.200 | 719.998 | 0.781 | .435 | [-1.415, 3.289] |
| Neutral x Externalizing | -1.624 | 1.200 | 719.998 | -1.353 | .176 | [-3.976, 0.728] |
| Angry x Externalizing | 0.341 | 1.200 | 719.998 | 0.284 | .776 | [-2.011, 2.693] |
| Fearful x Externalizing | -0.359 | 1.200 | 719.998 | -0.299 | .765 | [-2.711, 1.993] |
| Internalizing x Externalizing | -0.765 | 1.181 | 79.626 | -0.648 | .519 | [-3.079, 1.55] |
| Happy-face habituation deviation x Internalizing | 0.831 | 1.226 | 719.998 | 0.678 | .498 | [-1.572, 3.235] |
| Neutral-face habituation deviation x Internalizing | -1.131 | 1.226 | 719.998 | -0.922 | .357 | [-3.534, 1.273] |
| Angry-face habituation deviation x Internalizing | 0.780 | 1.226 | 719.998 | 0.636 | .525 | [-1.624, 3.183] |
| Fearful-face habituation deviation x Internalizing | -1.188 | 1.226 | 719.998 | -0.969 | .333 | [-3.592, 1.216] |
| Happy-face habituation deviation x Externalizing | -0.456 | 1.200 | 719.998 | -0.380 | .704 | [-2.808, 1.896] |
| Neutral-face habituation deviation x Externalizing | -1.062 | 1.200 | 719.998 | -0.885 | .376 | [-3.414, 1.29] |
| Angry-face habituation deviation x Externalizing | 0.482 | 1.200 | 719.998 | 0.402 | .688 | [-1.87, 2.834] |
| Fearful-face habituation deviation x Externalizing | 1.682 | 1.200 | 719.998 | 1.402 | .161 | [-0.67, 4.034] |
| Overall habituation x Internalizing x Externalizing | 0.238 | 1.136 | 90.000 | 0.209 | .835 | [-1.989, 2.464] |
| Happy x Internalizing x Externalizing | -0.196 | 0.789 | 719.998 | -0.248 | .804 | [-1.743, 1.351] |
| Neutral x Internalizing x Externalizing | -0.018 | 0.789 | 719.998 | -0.022 | .982 | [-1.564, 1.529] |
| Angry x Internalizing x Externalizing | -0.383 | 0.789 | 719.998 | -0.485 | .628 | [-1.929, 1.164] |
| Fearful x Internalizing x Externalizing | 1.319 | 0.789 | 719.998 | 1.672 | .095 | [-0.227, 2.866] |
| Happy-face habituation deviation x Internalizing x Externalizing | -0.457 | 0.789 | 719.998 | -0.579 | .563 | [-2.003, 1.09] |
| Neutral-face habituation deviation x Internalizing x Externalizing | -0.697 | 0.789 | 719.998 | -0.884 | .377 | [-2.244, 0.849] |
| Angry-face habituation deviation x Internalizing x Externalizing | -0.302 | 0.789 | 719.998 | -0.383 | .702 | [-1.849, 1.244] |
| Fearful-face habituation deviation x Internalizing x Externalizing | 0.472 | 0.789 | 719.998 | 0.599 | .550 | [-1.074, 2.019] |

Supplementary Table 12. Left amygdala: clinical, demographic, and activation differences by habituation type in treatment-referred youth

| Variable | Habituation | Sensitization | Test | Statistic | p | q |
| --- | --- | --- | --- | --- | --- | --- |
| **Age at T0** | 22.04 (2.23) | 21.40 (2.53) | t-test | t(82.03) = 1.28 | .202 | .292 |
| **IQ** | 83.00 (10.13) | 81.10 (11.14) | t-test | t(79.28) = 0.85 | .397 | .427 |
| **Adverse childhood experiences** | 4.11 (2.03) | 3.36 (1.64) | t-test | t(95.49) = 2.02 | .046 | .149 |
| **Delinquency score** | 34.54 (30.97) | 25.66 (27.69) | t-test | t(90.47) = 1.47 | .145 | .292 |
| **Internalizing symptoms** | 75.45 (22.76) | 71.24 (23.70) | t-test | t(86.5) = 0.88 | .379 | .427 |
| **Externalizing symptoms** | 73.71 (22.31) | 66.17 (23.09) | t-test | t(86.79) = 1.62 | .108 | .280 |
| **YPI affective** | 11.07 (4.12) | 10.52 (2.63) | t-test | t(92.42) = 0.8 | .427 | .427 |
| **Reactive aggression** | 11.95 (4.41) | 10.69 (4.66) | t-test | t(85.7) = 1.35 | .180 | .292 |
| **Proactive aggression** | 5.62 (4.60) | 4.60 (3.27) | t-test | t(95.76) = 1.3 | .198 | .292 |
| **Past-30-day cannabis use (days)** | 17.95 (12.74) | 11.46 (13.11) | t-test | t(84.91) = 2.43 | .017 | .106 |
| **Years of regular cannabis use** | 5.00 (3.71) | 3.22 (3.76) | t-test | t(85.62) = 2.29 | .024 | .106 |
| **Early amygdala activation** | 4.03 (15.18) | -4.77 (11.30) | t-test | t(96) = 3.29 | .001 |  |
| **Late amygdala activation** | -10.57 (11.87) | 8.09 (12.90) | t-test | t(84.3) = -7.33 | < .001 |  |
| **Late−Early change (Δβ)** | -14.60 (13.39) | 12.86 (13.98) | t-test | t(86.37) = -9.8 | < .001 |  |
| **Ethnic background** | 12 (21.43%) | 15 (35.71%) | Fisher's exact |  | .381 | .427 |
| Cape Verdean | 6 (10.71%) | 1 (2.38%) |  |  |  |  |
| Morroccan | 7 (12.50%) | 7 (16.67%) |  |  |  |  |
| Dutch | 10 (17.86%) | 4 (9.52%) |  |  |  |  |
| Other non-Western | 8 (14.29%) | 8 (19.05%) |  |  |  |  |
| Other Western | 2 (3.57%) | 0 (0.00%) |  |  |  |  |
| Surinamese | 10 (17.86%) | 6 (14.29%) |  |  |  |  |
| Turkish | 1 (1.79%) | 1 (2.38%) |  |  |  |  |
| **Primary problem cannabis** | 33 (58.93%) | 37 (88.10%) | Chi-square | χ²(1) = 10 | .002 | **.020** |
| Yes | 23 (41.07%) | 5 (11.90%) |  |  |  |  |

Supplementary Table 13. Right amygdala: clinical, demographic, and activation differences by habituation type in treatment-referred youth

| Variable | Habituation | Sensitization | Test | Statistic | p | q |
| --- | --- | --- | --- | --- | --- | --- |
| **Age at T0** | 21.85 (2.07) | 21.68 (2.66) | t-test | t(91.92) = 0.35 | .724 | .992 |
| **IQ** | 82.00 (9.77) | 81.98 (11.09) | t-test | t(91.36) = 0.01 | .992 | .992 |
| **Adverse childhood experiences** | 4.02 (1.79) | 3.56 (2.01) | t-test | t(94.71) = 1.2 | .235 | .509 |
| **Delinquency score** | 32.61 (29.72) | 29.50 (30.06) | t-test | t(91.91) = 0.5 | .615 | .992 |
| **Internalizing symptoms** | 73.38 (23.04) | 73.74 (23.68) | t-test | t(94.88) = -0.08 | .940 | .992 |
| **Externalizing symptoms** | 74.21 (22.12) | 66.80 (23.33) | t-test | t(94.99) = 1.61 | .112 | .509 |
| **YPI affective** | 11.35 (3.98) | 10.40 (3.10) | t-test | t(84.92) = 1.29 | .199 | .509 |
| **Reactive aggression** | 11.51 (4.23) | 11.36 (4.88) | t-test | t(94.4) = 0.16 | .871 | .992 |
| **Proactive aggression** | 5.74 (3.89) | 4.74 (4.26) | t-test | t(94.92) = 1.21 | .228 | .509 |
| **Past-30-day cannabis use (days)** | 17.81 (12.46) | 12.41 (13.49) | t-test | t(93.87) = 2.04 | .044 | .509 |
| **Years of regular cannabis use** | 4.84 (3.45) | 3.69 (4.11) | t-test | t(89.93) = 1.47 | .144 | .509 |
| **Early amygdala activation** | 5.05 (10.48) | -5.56 (9.62) | t-test | t(92.99) = 5.19 | < .001 |  |
| **Late amygdala activation** | -9.53 (10.57) | 4.87 (11.52) | t-test | t(94.95) = -6.42 | < .001 |  |
| **Late−Early change (Δβ)** | -14.58 (9.31) | 10.43 (10.76) | t-test | t(94.38) = -12.26 | < .001 |  |
| **Ethnic background** | 15 (31.91%) | 12 (24.00%) | Fisher's exact |  | .969 | .992 |
| Cape Verdian | 2 (4.26%) | 5 (10.00%) |  |  |  |  |
| Morroccan | 6 (12.77%) | 7 (14.00%) |  |  |  |  |
| Dutch | 7 (14.89%) | 7 (14.00%) |  |  |  |  |
| Other non-Western | 7 (14.89%) | 9 (18.00%) |  |  |  |  |
| Other Western | 1 (2.13%) | 1 (2.00%) |  |  |  |  |
| Surinamese | 8 (17.02%) | 8 (16.00%) |  |  |  |  |
| Turkish | 1 (2.13%) | 1 (2.00%) |  |  |  |  |
| **Primary problem cannabis** | 31 (65.96%) | 38 (76.00%) | Chi-square | χ²(1) = 1.19 | .275 | .511 |
| Yes | 16 (34.04%) | 12 (24.00%) |  |  |  |  |
| Values are M (SD) for continuous variables and n (%) for categorical variables. | | | | | | |
| p = nominal p-value; q = Benjamini–Hochberg adjusted p-value. Benjamini–Hochberg correction was used because these analyses were exploratory and involved multiple comparisons. This approach controls the false discovery rate while preserving more power than more conservative corrections. | | | | | | |
| Adjusted p-values were computed across demographic, clinical, and substance-use comparisons within each ROI, excluding ROI-derived activation variables (early, late, and Δβ), which are shown descriptively. | | | | | | |
| Because habituation subtype was defined from the same ROI time-course, ROI-derived activation differences should be interpreted as exploratory. | | | | | | |

| Supplementary table 14 | | | | |
| --- | --- | --- | --- | --- |
| Linear mixed effect model for Group (treatment-referred vs. control), Time (early vs. late) and condition (neutral, happy, sad, fearful, angry) for the left amygdala | | | | |
| **Effect** | **df1** | **df2** | **F** | **p** |
| Group | 1 | 122 | 2.082 | .152 |
| Time | 1 | 122 | 4.312 | .040 |
| Condition | 4 | 976 | 2.536 | .039 |
| Group x time | 1 | 122 | 1.103 | .296 |
| Group x condition | 4 | 976 | 0.727 | .574 |
| Time x condition | 4 | 976 | 4.120 | .003 |
| Group x Time x Condition | 4 | 976 | 4.086 | .003 |

| Supplementary table 15 | | | | |
| --- | --- | --- | --- | --- |
| Linear mixed effect model for Group (treatment-referred vs. control), Time (early vs. late) and condition (neutral, happy, sad, fearful, angry) for the right amygdala | | | | |
| **Effect** | **df1** | **df2** | **F** | **p** |
| Group | 1 | 122 | 0.860 | .356 |
| Time | 1 | 122 | 0.855 | .357 |
| Condition | 4 | 976 | 2.614 | .034 |
| Group x time | 1 | 122 | 0.198 | .657 |
| Group x condition | 4 | 976 | 0.590 | .670 |
| Time x condition | 4 | 976 | 3.959 | .003 |
| Group x Time x Condition | 4 | 976 | 4.182 | .002 |

| Supplementary table 16 | | | | | | | |
| --- | --- | --- | --- | --- | --- | --- | --- |
| Group differences in Δβ (Late − Early) per condition for the left amygdala | | | | | | | |
| **Condition** | **Contrast** | **Estimate** | **SE** | **df** | **95% CI** | **p** | **sig** |
| Happy | control - treatment-referred | -6.035 | 6.956 | Inf | [-19.669, 7.598] | .386 |  |
| Neutral | control - treatment-referred | -12.179 | 6.956 | Inf | [-25.813, 1.454] | .080 |  |
| Angry | control - treatment-referred | -0.594 | 6.956 | Inf | [-14.228, 13.039] | .932 |  |
| Fearful | control - treatment-referred | -16.703 | 6.956 | Inf | [-30.336, -3.07] | .016 | * |
| Sad | control - treatment-referred | 4.653 | 6.956 | Inf | [-8.98, 18.286] | .504 |  |

| Supplementary table 17 | | | | | | | |
| --- | --- | --- | --- | --- | --- | --- | --- |
| Group differences in Δβ (Late − Early) per condition for the right amygdala | | | | | | | |
| **Condition** | **Contrast** | **Estimate** | **SE** | **df** | **95% CI** | **p** | **sig** |
| Happy | control - treatment-referred | -1.410 | 6.29 | Inf | [-13.739, 10.919] | .823 |  |
| Neutral | control - treatment-referred | -5.580 | 6.29 | Inf | [-17.909, 6.749] | .375 |  |
| Angry | control - treatment-referred | 1.206 | 6.29 | Inf | [-11.123, 13.535] | .848 |  |
| Fearful | control - treatment-referred | -14.070 | 6.29 | Inf | [-26.399, -1.741] | .025 | * |
| Sad | control - treatment-referred | 8.151 | 6.29 | Inf | [-4.179, 20.48] | .195 |  |

| Supplementary Table 18 |  | | | |  |  |
| --- | --- | --- | --- | --- | --- | --- |
| MNI coordinates of local maxima activated the contrast “Face vs. Control” for the Whole brain ANOVA for the Face Processing task in a subgroup of individuals who did not regularly use cannabis ( n = 94). Results were cluster-corrected using p <0.001 as an initial threshold.  None of the other contrasts resulted in significant activation after FWE correction. | | | | | | |
| Area of activation | MNI Coordinates | | | Test statistic | | Cluster Size |
|  | x | y | z | *T* | |  |
| *Face vs. Control* |  |  |  |  | |  |
| Occipital_inf_R | 30 | -91 | 0 | 6.35 | | 89 |
| Precentral_R | 30 | 2 | 42 | 4.40 | | 109 |
